# Anti- and Pro-Oxidant Activity of Polyphenols Extracts of Syrah and Chardonnay Grapevine Pomaces on Melanoma Cancer Cells

**DOI:** 10.3390/antiox12010080

**Published:** 2022-12-29

**Authors:** Ylenia Spissu, Katarzyna Angelika Gil, Antonio Dore, Giulia Sanna, Giuseppe Palmieri, Andrea Sanna, Maurizio Cossu, Feten Belhadj, Boutheina Gharbi, Maria Barbara Pinna, Antonio Barberis, Carlo Ignazio Giovanni Tuberoso, Guy D’hallewin

**Affiliations:** 1 Institute of Sciences of Food Production (ISPA), 07100 Sassari, Italy; 2 Department of Life and Environmental Sciences, University of Cagliari, 09042 Monserrato, Italy; 3 Institute for Genetic and Biomedical Research (IRGB), 07100 Sassari, Italy; 4 IZS Sardegna SC Chimica, 07100 Sassari, Italy; 5 UTICA-Union Tunisienne de l’Industrie du Commerce et de l’Artisanat, Rue Ferjani Bel Haj Ammar, Cité Khadhra, Tunis 1003, Tunisia; 6 Laboratory of Research and Analysis Cantina Argiolas, 09040 Serdiana, Italy

**Keywords:** polyphenols, grapevine pomace, antioxidants, cyclic voltammetry, prooxidant activity, copper, melanoma cancer cells

## Abstract

**Simple Summary:**

In this paper the phenolic composition of Syrah and Chardonnay grape pomaces was studied to assess their antioxidant and prooxidant properties, and their effect on melanoma cancer cells.

**Abstract:**

The phenolic composition of Syrah and Chardonnay grape pomaces was studied to assess their antioxidant and prooxidant properties. Polyphenols were extracted by a "green" hydroalcoholic solvent (ethanol/water 1:1 *v*/*v*), and a detailed chemical and electrochemical characterization of the phenolic compounds was performed. The antioxidant and prooxidant capacity of the pomace was first studied by cyclic voltammetry (CV) and other reference analytical assays, then with biological tests on B16F10 metastatic melanoma cancer cells. Electrochemical data showed that, when a +0.5 V potential was applied, a low to moderate antioxidant capacity was observed. MTT test showed an increasing viability of melanoma cells, after treatments at low concentration (up to 100 μg/mL) and for a short time (6 h), but when cells were treated with higher doses of extract (≥250 μg/mL for 12/24 h), their viability decreased from 25 to 50% vs. control, depending on treatment time, dose, and extract origin. A stronger prooxidant activity resulted when 250 μg/mL of extract was combined with non-toxic doses of H_2_O_2_; this activity was correlated with the presence of copper in the extracts. This study shows the potential of winemaking by-products and suggests the opportunity to exploit them for the production of cosmeceuticals, or for combined therapies with approved anticancer drugs.

## 1. Introduction

Natural polyphenols are secondary metabolites of plants involved in defense against several types of stress. They act on multiple targets in pathways and mechanisms related to carcinogenesis, tumor cell proliferation, metastatic spread, and drug resistance [1]. Cancer-protective effects have been reported for people following a Mediterranean diet which is marked by a high content of plant polyphenols [2]. The role of these compounds as natural antioxidants to protect the damage by free radicals has long been recognized, nevertheless, under aerobic conditions, antioxidants generate superoxide radicals that dismutate to hydrogen peroxide (H_2_O_2_) which reacts with reduced metal ions and superoxide to form toxic reactive oxygen species (ROS); the overproduction of ROS has been implicated in the development of various chronic and degenerative diseases, including different cancers [3,4,5]. According to previous studies, polyphenols can act as antioxidants or prooxidants, depending on the concentration and the cellular environment [6,7,8]. Moreover, there are studies indicating that the antioxidant properties of polyphenols could be attributed to a chemopreventive action but not to therapeutic effects against tumors [9]. The antioxidant action of polyphenols is reported among different anticancer mechanisms, most of which implicate the modulation of gene expression and cellular signaling that leads to growth inhibition, apoptosis, or cell cycle arrest in cancer cells [10,11]. An immune system-dependent antitumor activity has been attributed to polyphenols in a melanoma mouse model [12], as well as a potential therapeutic role in melanoma metastasis has been claimed for various classes of phenolic compounds, non-flavonoids such as resveratrol and flavonoids such as proanthocyanidins, anthocyanins quercetin, and some catechins [13,14]. By contrast, much less consideration has been devoted to the research on the prooxidant effects of polyphenols on biological molecules [15,16], including the prooxidant anticancer activity of plant-derived compounds. The orientation of scientific production is now changing and, some authors have suggested looking for a common mechanism in the prooxidant action of polyphenols which involves intracellular copper mobilization [16,17]. Polyphenolic compounds in the presence of copper ions act as prooxidants, causing the breakage of DNA via ROS generation and leading to apoptosis [4].

For some time, the antioxidant activity of vegetable matrices has been investigated with analytical systems, mainly spectrophotometric or chromatographic, capable of indirectly measuring, with a single assay, the sum of the antioxidant activities of many molecules [18]. Compared with these, the electrochemical method has advantages which are extensively discussed in a recent review [19]. The direct electrochemical determination of antioxidants in plants or plant extracts [20,21,22] has been largely used since the ionization potential is the key factor that determines the efficiency of antioxidants [23,24], and because those molecules are easily oxidized on bare or nanostructured electrodes [25,26,27,28]. Cyclic voltammetry (CV) has been used for antioxidant capacity evaluation because the potential at which the oxidation starts enables the identification of the type of antioxidant involved, whereas the peak potential is an indicator of the antioxidant capacity [29]. Sun-Waterhouse et al. [30] estimated the antioxidant capacity in onions integrating the area under the peak up to 0.5 V; Pilijac-žegarac et al. [31] raised that threshold to 0.6 in tea infusions. More recently the same threshold has been lowered to 0.45 V in a study on Cabernet Sauvignon, Carménère, and Syrah pomaces; in the same study authors used CV to distinguish between antioxidant and prooxidant activity of pomace extracts obtained from *Vitis vinifera* L., assuming that polyphenols with oxidation potential lower than 0.45 V exert antioxidant activity, while those with oxidation potential greater than 0.45 V exert prooxidant activity [32]. The CV method was also applied to study the profile of flavonoids in onions [33], and to assess the total phenolics and the flavonols content in red and white wines [34]. When combined with HPLC-DAD analysis or mass spectrometry, CV has provided information on the contribution of phenolic compounds of mastic tree leaf extracts and citrus juices to antioxidant capacity [35,36]. 

Extracts containing polyphenols have been used for thousands of years in traditional eastern medicine. There is evidence that long-term consumption of moderate quantities of polyphenols present in red grapes and red wine can reduce the incidence of certain cancers [37]. Pomaces are byproducts of the winery and grape juice industry and their management represents an important environmental issue [38]. Great attention has been focused on this waste material because pomaces, that consist of skins, remaining pulp, seeds, and stalks, contain high amounts of health-promoting compounds, a high content of fiber and polyphenols that remain after the winemaking process [39]. The most abundant phenolic compounds in red wine pomaces are anthocyanins concentrated in the skin, while flavonols are more present in the seeds (56–65% of total flavonols) [40]. Phenolic acids, flavan-3-ols, flavonoids, and oligomeric procyanidins were identified in Chardonnay (white variety) pomaces [39]. Grape seeds extracts contain mainly the flavan-3-ols catechin and epicatechin, and procyanidins [41], but these compounds have also been isolated in skins. The total phenolic content of grape pomace extracts is usually well correlated to their antioxidant activity [42]. A greater concentration of phenolic compounds in the seeds than in the skins, and a good correlation with antioxidant activity, were observed for Brazilian grape varieties [43]. Spigno et al. [44] and Jara-Palacios et al. [45] also observed a high and significant correlation between antioxidant activity and total phenolic content in grape pomace samples.

In this paper, we have studied the anti- and prooxidant properties of extracts obtained from the winemaking process residual pomaces of Syrah and Chardonnay cultivars of *V. vinifera*. The pomaces came from countries of the western Mediterranean area, France, Tunisia, and Italy, that participate in the same BestMedGrape project [46] being interested in recycling winemaking waste. After a detailed chemical characterization, the antioxidant and prooxidant capacity of the extracts and their phenolic compounds were investigated by electrochemical tests and biological assays. Cyclic voltammetry was used to distinguish polyphenols that oxidize at low potential, from those with an ionization power too high to be properly considered antioxidants. Analytical tests were performed in parallel with biological assays: prooxidant anticancer activity was assayed by MTT viability and invasion tests on B16F10 metastatic melanoma cancer cells treated with pomace extracts, and with a combination of the latter with different concentrations of H_2_O_2_. The role of copper in the prooxidant activity was also investigated, since we assumed that residual copper from phytosanitary treatments, which are traditionally carried out on *V. vinifera*, may have influenced the response of cells to treatments with the extracts.

## 2. Materials and Methods

### 2.1. Chemicals and Reagents

#### 2.1.1. Analytical Assays

All the solvents and chemicals used in this study were of analytical grade. Methanol, ethanol, acetonitrile LC-MS grade, and 85% phosphoric acid were purchased from Merck (Darmstadt, Germany, Ethanol from Carlo Erba Reagenti (Milan, Italy). Standards of phenolic compounds were obtained from Extrasynthese (Genay, France), TransMIT (Giessen, Germany), and Merck-Sigma-Aldrich (Milan, Italy). Ultrapure water (18 MΩ·cm) was obtained with a Milli-Q Advantage A10 System apparatus (Millipore, Milan, Italy).

#### 2.1.2. Biological Assays

2-propanol BioReagent, thiazolyl blue tetrazolium bromide powder (MTT), Hydrogen peroxide solution, Glutamine 200 mM and penicillin-streptomycin solution were from Merck Life Science (Milan, Italy). The phosphate-buffered saline (PBS) solution was made using NaCl (137 mM), KCl (2.7 mM), Na_2_HPO_4_ (8.1 mM), and KH_2_PO_4_ (1.47 mM) from Merck Life Science and then adjusted to pH 7.4. Dulbecco’s Modified Eagle’s Medium (DMEM), fetal bovine serum (FBS), MEM non-essential amino acid 100X, and trypsin 0.25% EDTA solution were purchased by Euroclone (Milano, Italy).

### 2.2. Sample Collection and Preparation

Syrah and Chardonnay grape pomaces were supplied by wine farms located in Khanqat al Hajjaj, Grombalia (Tunisia), Cantina Argiolas, Serdiana (Italy), and INRA Pech Rouge, Gruissan (France). The Syrah pomaces were derived from plants cultivated on soils with different textures (Holocene sands with marl and clay in Italy; clay soils in Tunisia; sandy clay loam in France). At the laboratory, grape pomaces were rinsed with tap water to remove residual sugars, stems, and debris, drained, and move to a ventilated oven on air flow at 60 °C until a crushable material was obtained. The sample was then ground in a blade mill to achieve a fine powder and reduced to a particle size of < 425 microns by sieving. The recovered powder was freeze-dried to remove water traces, packed under vacuum, and stored at −20 °C until extraction.

### 2.3. Hydroalcoholic Extraction of Polyphenols

Lyophilized pomace samples (1 g) were transferred in 75 mL plastic tubes, added 25 mL of solvent (ethanol/water 1:1 *v*/*v*), sealed with a stopper, and shaken at 50 rpm for 5 h at room temperature in the dark. The liquid and solid phases were separated by centrifugation at 3220× *g* for 15 minutes and, subsequently, filtrated through a filter paper under vacuum. The filtrate was transferred in a vacuum roto-evaporator, where the ethanol evaporated at 40–45 °C. Finally, water was removed by freeze-drying. The dry residue was weighed, transferred in a sealed vial, and stored at −80 °C until analyses.

### 2.4. Identification and Quantification of Polyphenols in the Extracts

The total polyphenol content (TPC) was determined by the Folin–Ciocalteu method as previously reported [47], and the results were expressed as mg of gallic acid equivalents (GAE) per g of dry residue (dr). 

The quantitative analysis on targeted phenolic compounds was carried out using a modified HPLC-DAD method as described by Perra et al. [48] using an Agilent 1260 Infinity II HPLC system (Agilent Technologies, Cernusco sul Naviglio, Italy) fitted with a pump module G7111A, an autosampler module G7129A, and an Agilent G4212B photodiode array detector. The separation was obtained with a Kinetex EVO C18 column (150 × 4.60 mm, 2.6 μm, Phenomenex, Casalecchio di Reno, Italy) using 0.22 M phosphoric acid (solvent A) and acetonitrile (solvent B) as mobile phase, at a constant flow rate of 0.8 mL/min. The gradient (*v*/*v*) was generated decreasing from 100% solvent A to 80% in 20 min, to 70% in 35 min, to 0% in 45 min, and then remaining stable up to 50 min; finally, the gradient gets to 100% and stay stable 5 min before the following injection. The injection volume was 10 μL. The chromatograms and spectra were elaborated with an OpenLab V. 2.51 data system (Agilent Technologies, Cernusco sul Naviglio, Italy), and polyphenols were detected and quantified according to the main classes: anthocyanins at 520 nm, flavonols at 360 nm, hydroxycinnamic acids at 313 nm, hydroxybenzoic acids at 280 nm, and flavan-3-ols at 210 nm. Stock standard solutions were prepared in methanol and the working standard solutions were prepared in ultrapure water. The calibration curves for commercial standards were plotted with the method of the external standard, correlating the peak area with the concentration by means of the least-squares method, with a coefficient of determination (*r*^2^) > 0.998 in the range of 0.4–40 mg/L for all the compounds. Individual components were identified, or tentatively, by comparing the retention time and UV–Vis spectra of pure commercial standards or the UV–Vis spectra and the chromatographic profile described in the literature. Dry pomace extracts were diluted 1:25 *w*/*v* with a MeOH/H_2_O 80:20 *v*/*v* mixture for the HPLC-DAD analysis. The obtained solutions were filtered with a 0.45 μm CA syringe filter and diluted 1:2–1:5 *v*/*v* with 0.22 M H_3_PO_4_ before injection.

The quantitative evaluation of polyphenols was performed by dosing each phenolic compound with the corresponding analytical standard. When the commercial standard was not available, compounds were dosed using stock standard solutions of a proper reference compound: malvidin-3-*O*-glucoside, quercetin-3-*O*-glucoside, caftaric acid, gallic acid, and procyanidin B1, respectively. The total amount of phenolic compounds was obtained by summing the total amount of each phenolic class and results were expressed as mg/g of dr, and reported as mean ± SD.

### 2.5. Electrochemical Characterization and Antioxidant Activity Determination

The electrochemical characterization of the extracts and the AAox determination were performed by CV as previously reported [27,36] with some modifications. Measures were carried out by screen-printed sensors purchased by GSI Technologies (Burr Ridge, IL, USA), consisting of a 5 mm carbon working electrode (WE), an Ag/AgCl pseudo reference electrode (RE), and a carbon auxiliary electrode (AE). Currents were recorded by Quadstat, a commercial four-channel potentiostat (eDaQ Quadstat, e-Corder 410 and Echem software, eDAQ Europe Poland, Warsaw Poland). Cyclic voltammograms (CVs) were performed from −0.2 V to +0.8 V (vs. Ag/AgCl pseudo-RE) at a scan rate of 0.1 V/s. A first aliquot of 70 μL, containing only PBS (used as a supporting electrolyte), was deposited on the screen-printed WE with a graduated micropipette in order to obtain a baseline. Once the baseline current was recorded, the PBS drop was dried with absorbent paper without touching the surface of the sensor, and 70 μL aliquots of a 2 mg/mL pomaces extract solution were deposited on the sensor surface (the experiment was performed in triplicate) thus obtaining the corresponding CV pattern. In order to provide a quantitative comparison among the CV patterns of extracts of different origin, the voltammograms were integrated and the area under curve (AUC) was calculated at +0.5 V and +0.8 V and expressed in microcoulombs (μC). The redox potential of +0.5 V is used as a threshold to detect the antioxidant activity of pomace extracts, and +0.8 V to calculate the TPC, in accordance with previous studies [26,30,31]. As already reported [35,49,50], oxidation potentials higher than +0.5 V refer to polyphenols with low reducing power which, in this work, were not accounted as antioxidants.

Then, to determine the redox potential of the most represented polyphenols in the extracts, (those represented in quantities ≥ 0.75 mg/mL according to the HPLC-DAD analysis), the CVs of the relative standard molecules were carried out. CVs of increasing GA concentration (from 0.1 to 3 mM) were recorded, and the mathematical parameters (equation and *r*^2^) of the reference calibration curve were calculated (Figure S1 in Supplementary Material). The antioxidant capacity of the standard molecules was determined by referring to this calibration curve, at the threshold value of +0.5 V, and expressed in terms of GA equivalent millimoles.

### 2.6. Antioxidant Activity Determination with CUPRAC and DPPH Reference Methods

CUPRAC and DPPH^•^ assays were performed according to Bouzabata et al. [51]. For the CUPRAC assay, 100 μL of diluted sample was dissolved in a mixture of 500 μL of 10 mM CuCl_2_ solution in water, 500 μL of 7.5 mM neocuproine solution in methanol, and 500 μL of 1.0 M CH_3_COONH_4_ buffer at pH = 7.0. After an incubation period of 30 min in the dark, absorbance at 450 nm was measured. Quantitative analysis was performed according to the external standard method using 0.1–2 mmol/L FeSO_4_ and results were expressed as mmol/g of Fe^2+^ per g of dr. For the DPPH^•^ assay, 50 μL of diluted sample was dissolved in 2 mL of 0.06 mmol/L DPPH^•^ in methanol. Then, spectrophotometric readings were carried out at 517 nm after an incubation period of 60 min in the dark. A calibration curve in the range of 0.02–1.0 mmol/L was prepared for Trolox, and the data were expressed as the Trolox equivalent antioxidant capacity (TEAC mmol/g dr).

### 2.7. Determination of Prooxidant Activity of Polyphenols on Melanoma Cancer Cells

The prooxidant activity of polyphenols’ pomace extracts was determined on B16F10 murine melanoma cell lines, and on human fibroblasts used as normal control.

#### 2.7.1. Viability Assays 

B16F10 murine melanoma cell lines and human fibroblasts were obtained from ATCC and were maintained on DMEM supplemented with 10% FBS, 1% penicillin/streptomycin, 2 mM Glutamine, and 1X MEM non-essential amino acid at 37 °C under 5% CO_2_ and 95% humidity.

B16F10 cells were plated in 96 wells at a concentration of 1 × 10^4^/100 μL and three different experiments were carried out: (1) a time-dose response test for 6, 12, and 24 h with increasing concentrations (1-10-50-100-250-375-500 μg/mL) of Syrah or Chardonnay pomace extract from Italy, France and Tunisia; (2) treatments with increasing concentration of H_2_O_2_ (1-10-50-100-200-300-400-500 μM) for 24 h; 3) a combined treatment with Syrah 250 μg/mL + H_2_O_2_ 10-50-100 μM, or Chardonnay 250 μg/mL + H_2_O_2_ 10-50-100 μM for 24 h.

Fibroblasts were plated in 96 wells at a concentration of 2 × 10^4^/100 μL and two different experiments were carried out: (1) a dose-response test for 24 h with increasing concentrations (1-10-50-100-250-375-500 μg/mL) of Syrah or Chardonnay extract; (2) treatments with increasing concentration of H_2_O_2_ (1-10-50-100-200-300-400-500 μM) for 24 h.

At the end of the experiments, cells were incubated with 100 μL of MTT (0.5 mg/mL), and the cultures were allowed to incubate at 37 °C for 3 h. The MTT was removed and the formazan crystals were dissolved in 100 μL of 2-propanol. The color was read at 570 nm using a microplate reader (Sunrise™ Absorbance Reader—TECAN, Hombrechtikon, Switzerland). The percentage of cell growth was calculated by normalizing the absorbance of treated cells to the corresponding control. All the experiments were performed in triplicate.

#### 2.7.2. Invasion Test

B16F10 cells were plated in 24 wells at a concentration of 2 × 10^5^/mL. After 24h, a wound was performed using a tip, and the cells were treated with 250 μg/mL of Syrah or Chardonnay from Italy, France, and Tunisia for 24 h. At the end of the experiment, the cells were washed twice with PBS, and a snapshot image to verify wound closure was taken with an inverted microscope. The area of the wounds was calculated using the ImageJ software. All the experiments were performed in triplicate.

### 2.8. Cu Measurement

Copper was determined by an inductively coupled plasma mass spectrometry (ICP-MS/MS), in compliance with US EPA 6020B. The analytical procedure involved acid digestion of the powder sample (0.5 g) in a glass vessel with 5 mL of ultrapure nitric acid, 70% (J.T. Baker, Phillipsburg, NJ, USA). Treatment was carried out on the Discover SP-D microwave digestion system (CEM Corp., Charlotte, NC, USA) at a power of 600 W and a temperature fixed at 200 °C. The digestion solution was diluted to 50 mL with ultrapure water MILLI-Q^®^ Quantum^®^ TEX (from Merk, Darmstadt, Germany), and, before instrumental analysis, a second dilution of the 5 mL to 10 mL with the 2% solution of nitric acid. The liquid samples were analyzed directly after dilution of 0.5 mL of sample to 5 mL with the 2% solution of nitric acid.

The instrumental analysis was performed with an inductively coupled plasma mass spectrometer ICP-MS/MS (Agilent 8800 QQQ, Santa Clara, CA, USA) equipped with a collision cell and two quadrupole mass analyzers. ^63^Cu was used as a quantification isotope and ^74^Ge as an internal standard element to compensate for the matrix effect and signal drift. For each batch of samples, a method blank was carried throughout the entire sample preparation and analytical process. The calibration curve was verified at the start of each analytical batch using the initial calibration verification (ICV) with a different lot standard, while the instrumental sensitivity was verified using the continuous calibration verification (CCV) at or near midrange. Laboratory was intercalibrated through successful participation in internationally organized proficiency tests. The LOQs testing was 0.010 mg/kg for Cu. The quality control of the data was verified and controlled using Certified Reference Materials Rye Grass ERM-CD281 (10.2 ± 0.5 mg/kg). The method is accredited according to UNI EN ISO 17025/2017.

### 2.9. Statistical Analysis

Statistical analysis was performed by GraphPad Prism 5 for Windows software (GraphPad Software, Inc., La Jolla, CA, USA). Phenolics content of pomace extracts was expressed as mg/g of dr. AAox was expressed as micromoles equivalents of gallic acid/g of dr. For analytical tests, a one-way ANOVA was performed to compare results obtained with different analytical methods, using a unifactorial complete randomized block design. Mean comparisons were calculated by Fisher’s least significant difference (LSD) test at *p* ≤ 0.05.

Where not otherwise specified, biological tests were repeated three times. A one-way ANOVA was performed to highlight significant differences among treatments. The Student–Newman–Keuls (SNK) test was used to separate the mean values (*p* ≤ 0.01). The mean value ± standard deviation (SD) was reported in the figures.

## 3. Results

### 3.1. Chemical Characterization of Pomace Extracts

**Figure 1:**
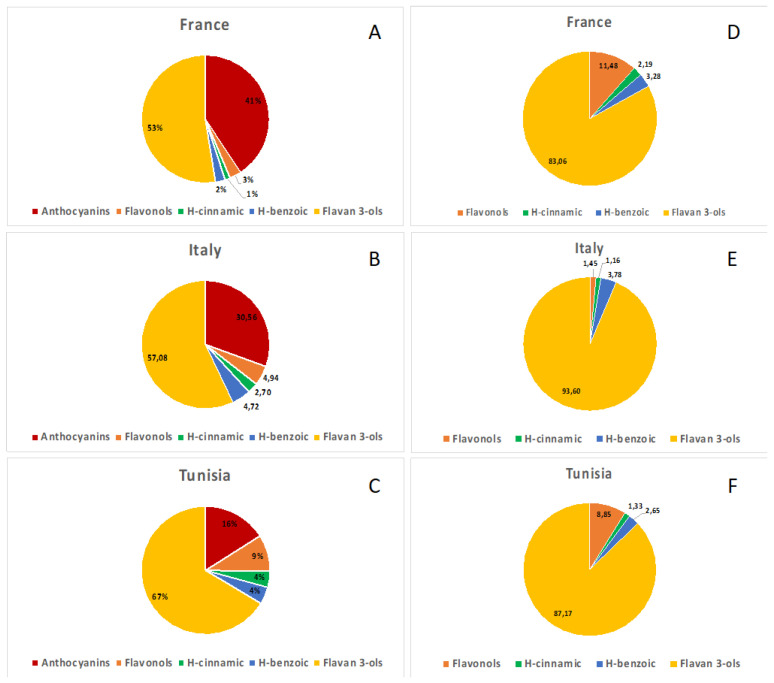
Pie charts of phenolic compounds of analyzed French, Italian, and Tunisian pomace extracts. The different classes of polyphenols are represented as % in figures (**A**–**C**) for Syrah, and in (**D**–**F**) for Chardonnay.

The qualitative HPLC-DAD characterization of polyphenolic compounds was performed according to their typical UV–Vis absorbance spectra and the comparison with pure standards or literature data [52]. The analysis of the three Syrah pomace extracts showed the presence of anthocyanins, flavonols, hydroxycinnamic and hydroxybenzoic acids, and flavan-3-ols, detected at 520, 360, 313, 280, and 210 nm, respectively; all the above-mentioned polyphenols classes, except anthocyanins, were also detected in Chardonnay pomace extracts (Figure 1). 

Syrah pomace extracts resulted to be richer (60.90, 44.50, and 36.92 mg/g dr for French, Italian, and Tunisian, respectively) in phenolic compounds than Chardonnay varieties (18.28, 34.44, and 22,62 mg/g dr for French, Italian, and Tunisian, respectively) (Tables S1 and S2 and Figure S1). The French Syrah variety was ca. 1.5 times more concentrated than the pomace extracts of the other two countries. In the case of Chardonnay, the Italian pomace extract is the richest in polyphenols, ca. 1.5 times higher than French and Tunisian, which contain similar amounts.

In Syrah, the fraction of the anthocyanins accounted for about 30% of all dosed phenolic compounds, varying from 16% in the Tunisian extract to 41% in the Italian one. The French sample showed the highest amount (24.77 ± 0.40 mg/g dr), followed by the Italian (13.55 ± 0.28 mg/g dr) and the Tunisian ones (5.96 ± 0.09 mg/g dr). Among the 13 detected anthocyanins, the most representative were malvidin-3-*O*-(*p*-coumaroyl)glucoside (2.49–10.05 mg/g dr) and malvidin-3-*O*-glucoside (1.11–6.43 mg/g dr), which accounted for 65.5% of all anthocyanins detected in three Syrah pomace samples. Moreover, malvidin-3-(acetyl)glucoside and peonidin-3-*O*-(*p*-coumaroyl)glucoside were detected in relatively high amounts. 

Seven different flavonols were identified in the Syrah and Chardonnay pomace samples, quercetin and its -3-*O*-glucoside, galactoside, and glucuronide derivatives, kaempferol, kaempferol-3-*O*-glucoside, and isorhamnetin. They accounted for about 5.2% and 6.1% of all phenolic compounds found in Syrah and Chardonnay, respectively, with the Tunisian Syrah and the Italian Chardonnay that showed the highest (3.44 ± 0.03 mg/g dr) and the lowest (0.54 ± 0.01 mg/g dr) amount.

Hydroxycinnamic and hydroxybenzoic acids were detected in all investigated pomace extracts. The cinnamic acid derivatives accounted for 2.4 and 1.4% of all polyphenols in the extracts of red and white varieties respectively, while benzoic acids accounted for 3.5% in both. French and Italian Syrah samples were ca. 2.5 times richer than Chardonnay equivalents, while Tunisian Syrah was ca. 5 times more than its white equivalent. Caftaric acid was the most representative hydroxycinnamic acid in both varieties. The hydroxybenzoic acids are more represented in Syrah than in Chardonnay, and in particular in the two Italian varieties. The gallic acid and, only in Syrah, the syringic acid, are the most represented phenolics of this class. 

Flavan-3-ols were highly detected in all samples. On average, they accounted for 59% and 88% of all detected phenolic compounds of Syrah and Chardonnay samples, respectively. The main representatives, both in Syrah and Chardonnay extracts, were the dimers (+)-catechin and (−)-epicatechin. In Syrah, the highest amount of flavan-3-ols was detected in the French pomace sample (6.19 ± 0.35 and 5.48 ± 0.16 mg/g dr of catechin and (−)-epicatechin, respectively), while in Chardonnay, the richest was the Italian sample (7.98 ± 1.08 and 7.83 ± 0.02 mg/g dr, respectively). In all samples, relatively high amounts of procyanidin B2 were also detected. The highest quantity of this monomer was found in the French Syrah (4.34 ± 0.20 mg/g dr) and in the Italian Chardonnay (2.79 ± 0.67 mg/g dr) pomace samples.

### 3.2. Electrochemical Characterization and Antioxidant Activity Determination

The electrochemical analysis of the pomaces’ samples was performed in order to establish the magnitude of their antioxidant capacity, and to determine the contribution of the different polyphenols to the activity of each extract. 

**Figure 2:**
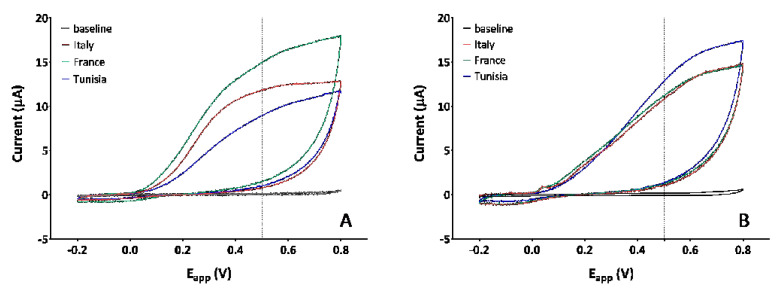
Cyclic voltammograms, with a scanned potential range (E_app_) comprised between −0.2 V and +0.8 V vs. carbon pseudoreference, in the absence (PBS black line) and in the presence of 2 mg/mL of Italian (red line), French (green line), and Tunisian (blue line) pomace extracts of Syrah (**A**), and Chardonnay (**B**).

Figure 2 shows the cyclic voltammograms of Syrah (Figure 2A) and Chardonnay (Figure 2B) pomace extracts from different origin.

CVs were obtained in the potential range −0.2 V +0.8 V in order to cover all groups of antioxidant compounds. All the voltammograms split from the baseline at around +0.1 V, indicating the presence of polyphenols with low redox potential, both in Syrah and Chardonnay; as the applied potential increases, the shape of the French and Italian Syrah voltammograms becomes more rounded between 0.25 and 0.4 V, indicating a large polyphenol component that ionizes in that potential range. That component is not present, or is less represented, in the Tunisian Syrah and in all three Chardonnay extracts. 

Different shapes correspond to different AUCs which are reported, both at 0.5 V and 0.8 V, in Table 1. The AUC_0.8_ (AUC values at +0.8V) values estimate the total polyphenols content, while AUC_0.5_ (AUC values at +0.5V) values refer to AAox. 

Looking for a correlation between the electrochemical data and the total polyphenols or the antioxidant activity of the extracts, it was found that, in Syrah: (i) there is a good correlation between AUC_0.8_ and TPC (*R*^2^ = 0.976), as well as between AUC_0.8_ and HPLC-DAD (*R*^2^ = 0.991); (ii) there is a low correlation between AUC_0.5_ and the values obtained by DPPH or CUPRAC; (iii) there is a good correlation between AUC_0.5_ and TPC (*R*^2^ = 0.969); (iv) there is a low correlation between AUC_0.5_ and the two most represented classes of phenolic compounds (both with anthocyanins and flavan 3-ols, and with the sum of the two). Differently, in Chardonnay: (i) no positive correlation was found between AUC values and TPC or HPLC-DAD; (ii) a low correlation was found between AUC_0.5_ and the values obtained by DPPH and CUPRAC; (iii) there is a moderate correlation between flavan 3-ols and AUC_0.5_ (*R*^2^ = 0.772), as well as between flavan 3-ols and AUC_0.8_ (*R*^2^ = 0.743).

In order to better understand the contribution of different polyphenols to the AAox, the CVs of 1 mM standard of the most represented polyphenols in the extracts, (those represented in quantities ≥ 0.75 mg/g dr according to the HPLD-DAD analysis) were carried out, and the redox potential was extrapolated (Table 2). The AAox of each standard (***x***) was expressed in equivalent millimoles of GA, on the basis of Equation (1), extrapolated from the calibration curve reported in Figure S2 in Supplementary Material:

*y* = 8.005*x* + 2.358 (1)

where ***y*** is the current recorded by ***x*** mM gallic acid solution at +0.5 V.

Table 2 reports the contribution of each polyphenol to the total AAox obtained by multiplying the ***x*** values for the relative molar concentration in the extract.

The results in Table 2 refer to standard molecules and, obviously, cannot take into account synergies or antagonisms among molecules in the phytocomplexes extracted from pomaces. Data only show the potential contribution of each polyphenol in comparison to the others. This contribution is a function of the redox potential and the concentration of each molecule in the extract. It is clear that flavan 3-ols provide the greatest contribution in both Syrah and Chardonnay. But it is equally clear that, among the anthocyanins (only in Syrah), peonidin-3-*O*-glucoside (0.75 mg/g dr in the French extract) which begins to oxidize at +0.23 V, provides a much greater contribution than malvidin-3-*O*-glucoside (6.43 mg/g dr in the French extract) which starts oxidizing at +0.55 V. 

### 3.3. Prooxidant Activity of Polyphenols on Melanoma Cancer Cells

**Figure 3:**
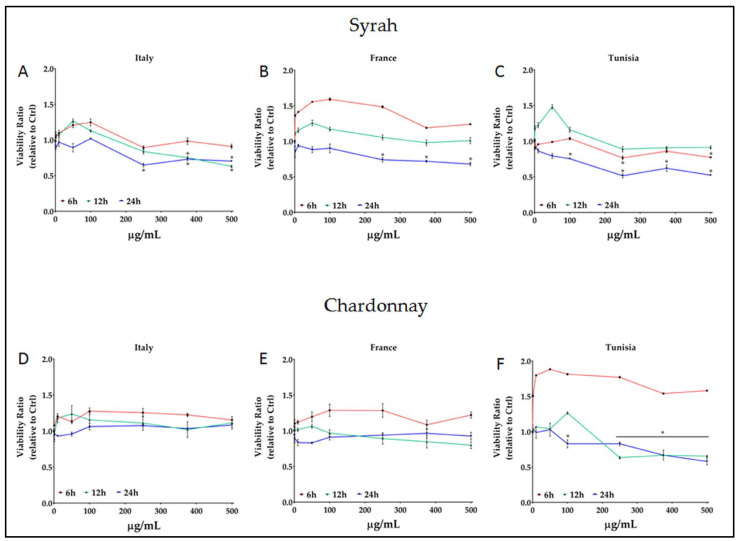
Effect of Syrah and Chardonnay pomace extracts on viability of B16F10 cancer cell lines. * = *p* ≤ 0.01 vs. control.

The results of the time–dose response MTT test with increasing concentrations of Syrah or Chardonnay pomace extracts on B16F10 cells are reported in Figure 3.

The data on the graph showed that at low concentrations (up to 100 μg/mL) and with reduced treatment time (6 h), there was an increase in cell viability: this was for both Syrah and Chardonnay, regardless of their origin. As the treatment time and the extract dose increased, a reversal of this trend was observed: this inversion is limitedly observable, after 12 h of treatment, only in the Italian Syrah (the viability decreased from 15 to 35% compared to the control, with concentrations from 250 to 500 μg/mL) and in the Tunisian Chardonnay (about 35% less, with concentrations from 250 to 500 μg/mL). After 24 h, the effectiveness of the treatments was significantly higher, with the following differences: a reduction in cell viability between 25 and 35% can be observed with the Italian and French Syrah samples, and between 35 and 50% with the Tunisian one, with an extract dose of 250 μg/mL or more; treatments with the Italian and French Chardonnay appeared ineffective, while the Tunisian pomace extracts reduced the viability of cancer cells by 17.4, 33.5, and 42.3% with 250, 375, and 500 μg/mL, respectively.

The effects of 24 h treatments were also studied on fibroblasts, used as a normal control, and shown in Figure S3 in Supplementary Material. No variation in cell viability was observed when the fibroblasts were treated with Tunisian Syrah, while a reduction of about 20% was seen with the Italian and French Syrah. On the other hand, the Italian and French Chardonnay extracts had no effect on fibroblasts, while the Tunisian one caused a 20 to 25% viability reduction with doses from 250 μg/mL upwards.

According to Floris et al. [53], a 20% reduction in viability is the threshold to consider a treatment to be effective. In our work, such a reduction was obtained with 250 μg/mL of extract, a dose that did not induce toxic effects on fibroblasts. Italian, French, and Tunisian Syrah extracts showed a reduction of 33.7, 26.1, and 49.1%, respectively, whereas Chardonnay extracts did not reach the threshold (Table 3) even at higher concentrations, with the exception of the Tunisian Chardonnay as reported above. In order to calculate how effective a 250 μg/mL dose was, compared to a reference oxidative stress inducer such as H_2_O_2_, a dose-response test with an increasing quantity of H_2_O_2_ was carried out both on B16F10 cells (Figure S4A ) and fibroblasts (Figure S4B), and a calibration curve (Figure S4C) was obtained. So, the pro-oxidant activity of 250 μg/mL of each pomace extract was calculated as μmoles equivalents of H_2_O_2_ (Table 3).

#### 3.3.1. Prooxidant Activity of Combined Treatment with Polyphenols and H_2_O_2_ on Melanoma Cancer Cells

**Figure 4:**
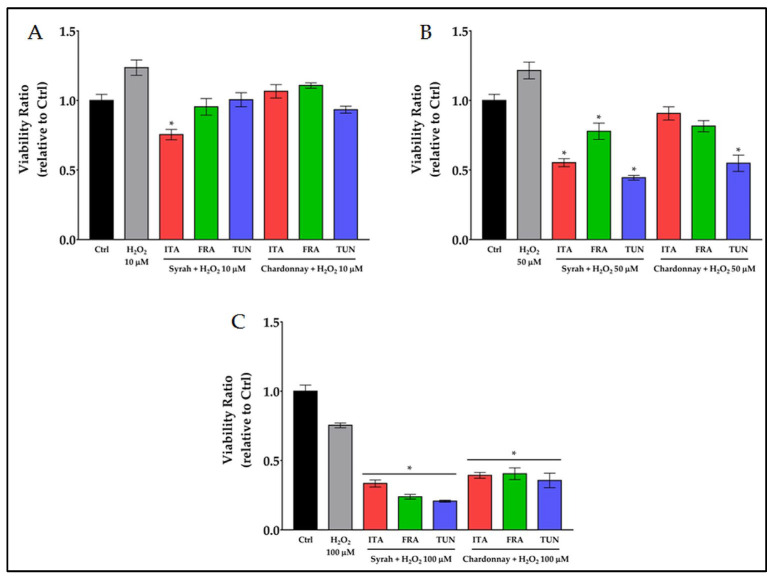
Prooxidant activity of 24 h combined treatments with 250 μg/mL pomace extracts and low concentrations (10, 50, and 100 μM, subfigure (**A**–**C**) respectively) of H_2_O_2_ on B16F10 cancer cell. * = *p* ≤ 0.01 vs. H_2_O_2_.

Since we observed that the viability of cancer cells was higher than the control when a moderate oxidative stress was induced (up to 100 μg/mL of polyphenols extracts), while it was reduced when the stress increased (over 250 μg/mL of polyphenols extracts), the prooxidant effect of combined treatments with 250 μg/mL pomace extracts and low concentrations of H_2_O_2_ was studied (Figure 4).

Ten, 50, and 100 μmoles/L are the concentrations of H_2_O_2_ that did not induce any toxic effect on fibroblasts (Figure S3B ). When 10 μM H_2_O_2_ was combined with 250 μg/mL of Syrah or Chardonnay extract, no prooxidant effect was observed compared to the positive control (H_2_O_2_) with the only exception of the Italian Syrah sample; when 50 μM H_2_O_2_ was combined with the extract, a significant reduction in viability was observed for Syrah and the Tunisian Chardonnay; when the dose of H_2_O_2_ increased to 100 μM, the reduction in viability was always significant both for Syrah and Chardonnay. In Table 4, the prooxidant effect of the combined treatment of 250 μg/mL extracts with 50 and 100 μM H_2_O_2_, was reported as a reduction in viability (%) vs. control; on the right side of the table shows the μmoles of H_2_O_2_ that would be necessary, if used alone, to obtain the same prooxidant effect.

The reduction in viability induced by the combined treatments varies from 45 to 80% with Syrah extracts and from 45 to 65% with Chardonnay extracts. This prooxidant effect on cancer cells was obtained with doses of H_2_O_2_ that did not have a toxic effect on fibroblasts. The same effect, using only hydrogen peroxide, could have been obtained only with cytotoxic doses for healthy cells (over 100 μM).

#### 3.3.2. Quantification of Copper (Cu) in the Syrah and Chardonnay Extracts

Copper has been used for over a century in agriculture: cupric products, in the form of various compounds and formulations, are a classic of the phytosanitary defense of vines to stop the spread of fungal and bacterial diseases. High copper levels in cancer cells were suggested to be a potential target for selective antitumoral action of plant polyphenols, because of their large availability and their null or low toxicity on fibroblasts [54]. For this reason, it was investigated whether and how much copper was left in the Syrah and Chardonnay extracts at the end of the extraction process of polyphenols from the pomaces: 201, 96, and 225 Cu ng/g dr were found in the Italian, French, and Tunisian Syrah extracts, respectively, and 112, 89, and 45 Cu ng/g dr were found in the Italian, French, and Tunisian Chardonnay extracts, respectively. A high correlation (*R*^2^ = 0.979) between Cu content and prooxidant activity was found in Syrah but not in Chardonnay.

#### 3.3.3. Effect of Syrah and Chardonnay Extracts on Invasion Capacity of Melanoma Cancer Cells

**Figure 5:**
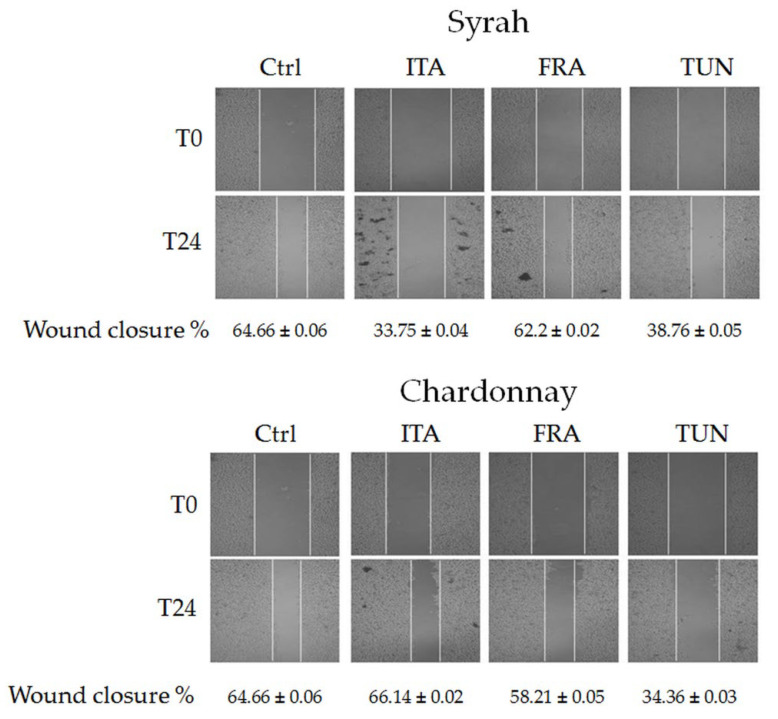
Snapshot microscopy images of wound closure (%) of untreated (Ctrl) and treated with 250 μg/mL of Syrah or Chardonnay from Italy, France, and Tunisia for 24 h B16F10 cells.

Invasion occurs when tumor cells disseminate from the primary tumor to colonize distant organs [55]. The capacity of the Syrah and Chardonnay extracts to limit the invasion ability of B16F10 cells is shown in Figure 5.

Once the monolayer was obtained and the wound created, the cells converged to close it. After 24 h, a closure of 64.66% of the wound was observed in the control. The treatment with Italian and Tunisian Syrah extracts limited the wound closure to 33.75 and 38.76%, respectively, while French Syrah did not have a significant effect. The Italian and French Chardonnays have been proven to be ineffective too, while the French Chardonnay resulted in a 34.36% closure.

## 4. Discussion

The objective of this study was to investigate the antioxidant and prooxidant properties of Syrah and Chardonnay pomace extracts. It was not the intention of this work to compare the properties of the two varieties, but even the comparison between extracts of different origins was affected by factors that could not be controlled at the origin. The original grapes were grown in different soil and climatic conditions and subjected to different winemaking processes. So, the quantitative and qualitative distribution of polyphenols has shown significant differences across varieties and among pomaces of different origin. This is the result of a lack of standardized protocols for processing waste; the needs of primary processing, the winemaking, did not take into account the future characteristics of the waste by-products. Nevertheless, we have found statistically similar anti- and prooxidant properties within the same variety.

### 4.1. Extraction and Characterization

The first step in defining the anti- and prooxidant properties of the pomace extracts was the quantitative and qualitative chemical characterization. The results obtained in this study find comparisons in the literature, even though a large difference in the extraction methods should be taken into account [44,56]. In accordance with recent studies [23], our choice fell on a "green" ethanol/water extractant mixture. It was a healthy and eco-friendly choice because the mixture of 50% ethanol and 50% water is not toxic to humans, is sustainable in terms of costs and safety for an industrial transformation process, and ensured a good yield of polyphenols without residual ethanol in the extract. This should be an essential condition, since any toxic residues in the extracts can alter the cell viability tests and the correct determination of the prooxidant capacity.

Chromatography for the identification and quantification of phenolic compounds was preferred in this work due to its robustness and widespread application. The HPLC analysis of methanolic extracts of Syrah cultivated in Spain showed the presence of different phenolic compounds in marcs (pomaces), stalks, and dregs [57]. Gallic acid, *p*-OH-phenethyl alcohol, syringic acid, and epicatechin were identified in the marcs. Epicatechin was ca. 5–19 times more concentrated than the other compounds (21.74 mg/L); a trend that was not observed in all Syrah samples investigated in our study. Mediterranean grape pomace, seed, and skin extracts were studied by Ky and Teissedre [56]. The analyses of two different French Syrah extracts, water (for edible extract) and 70% hydro-alcoholic solution (for nutraceuticals or cosmetic formulations), allowed to identify anthocyanins and flavan-3-ols. The use of the 70% hydro-alcoholic solution permitted a better extraction of polyphenols (particularly 3.2-fold more anthocyanins) than the water alone. The most represented anthocyanins were 3-*O*-monoglucosides of delphinidin, cyanidin, petunidin, peonidin, and malvidin, which is a similar trend to our findings, where the main anthocyanins detected were malvidin-3-*O*-glucoside and malvidin-3-*O*-(6″-*O*-coumaroyl)-glucoside. Lingua et al. [58] analyzed the pomaces and the wine extracts of several grape cultivars grown in Argentina, including the Syrah, and phenolic acids, flavonols and anthocyanins were identified. The three main anthocyanins were malvidin derivatives (142.22 ± 10.15, 195.01 ± 16.59, and 238.94 ± 4.75 mg/kg for malvidin-3-glucoside, malvidin-3-acethylglucoside, and malvidin-3-coumaroylglucoside, respectively), similar to what observed in our study. Among flavonols, quercetin and its 3-*O*-glucoside were detected in the highest amounts, while caftaric acid, coutaric acid, and ethyl gallate were detected in Syrah pomaces as the main non-flavonoid compounds. Regarding the pomace samples from the white Chardonnay variety, the HPLC-DAD analysis of the extract from Chile allowed to identify a range of different hydroxybenzoic acids and flavan-3-ols [59]. In the first class of phenolic compounds, gallic and protocatechuic were detected, and the main flavan-3-ols were procyanidin B1-B4, (+)-catechin, and (−)-epicatechin. A similar trend concerning flavan-3-ols was observed compared to our study: it was noticed that dimers were detected in higher amounts than monomers. However, the findings of Cerda-Carrasco et al. [59] showed significant differences in quantities of (+)-catechin and (−)-epicatechin, which was not observed in our research. The analysis of seeds and skins after aqueous and/or organic solvent extractions of Chardonnay grape varieties grown in Sardinia (Italy) allowed to identify a range of different phenolic compounds [60]. Among them were hydroxybenzoic acids or their derivatives (gallic acid, 3,4-di-OH benzoic acid, vanillic acid, ethyl gallate, ellagic acid), hydroxycinnamic acid (*p*-coumaric acid), flavan-3-ols and their derivatives (procyanidin B1, B2, catechin, epicatechin, epicatechin gallate), and flavonols (rutin, quercetin-3-*O*-glucoside, kaempferol-3-*O*-glucoside, quercetin, and kaempferol). Lu and Foo [39] identified different polyphenolic compounds in ethanolic extracts of Chardonnay grape pomace from New Zealand. These compounds were gallic acid and its *β*-glucopyranosides, trans-caftaric acid and 2-hydroxy-5-(2-hydroxyethyl)phenyl-*β*-glucopyranoside, *trans*- and *cis*-coutaric acids, procyanidin B1, catechin, epicatechin, astilbin, quercetin 3-glucuronide, quercetin 3-glucoside, kaempferol 3-galactoside, engeletin, and kaempferol 3-glucoside. Furthermore, in Spanish Chardonnay, skin and seed samples analyzed by Rodríguez Montealegre et al. [61] were found *cis*- and *trans*-caftaric acid, *cis*- and *trans*-coutaric acid, *trans*-fertaric acid, protocatechuic acid, catechin, epicatechin, epicatechin gallate, procyanidin B1-B4, quercetin glucuronide, quercetin glucoside, kaempferol glucoside, and isorhamnetin glucoside.

### 4.2. Polyphenols and Their Antioxidant Properties

The determination of TPC, as well as the determination of the antioxidant capacity of the extracts, is routinely carried out using toxic reagents (Folin–Ciocalteu, DPPH, CUPRAC, and most of the traditional assays involve the use of toxic solvents). The use of the aforementioned methods in this work is to offer, to those who legitimately use them [56], a term of comparison. Nevertheless, our choice fell on cyclic voltammetry since it can provide, with only one measure, a direct evaluation of antioxidant activity (AUC_0.5_) and of total polyphenols content (AUC_0.8_). This electrochemical approach has proven to be efficient in complex matrices such as red and white wines [34,62,63] or by-products of wineries [32,45]. Moreover, it is simple, fast, inexpensive, sensitive, reliable, and, most important, free from the interferences which lead colorimetric assays to overestimate the results [20]. This overestimation could be the reason why the correlation we found between AUC_0.5_ values and DPPH and CUPRAC assays was low, both in Syrah and Chardonnay. The AUC_0.5_ values we deduced from the CVs of pomaces are in the same order of measure as those of Jara-Palacios et al. –Similar results were obtained by those authors in red and white wines, where the Q_500_ (which corresponds to our AUC_0.5_) was used as a measure of the concentration of the total phenols and to estimate the concentration of the low formal potential antioxidants. The measures obtained by CV were considered a good estimation of the concentration of the more reactive antioxidants in the wines, and they were 4–5 times lower than Folin–Ciocalteu values. Furthermore, the lack of a high correlation between the AUC_0.5_ and the content of the most represented classes of phenolic compounds, anthocyanins and flavan-3-ols in Syrah, and flavan 3-ols in Chardonnay, should not surprise, as also found by Piljac et al. [64]: the quantitative analysis of the single molecules cannot take into account the synergies and antagonisms between them and, in addition, many of the most represented molecules in the examined extracts can offer a minimal contribution to the antioxidant activity. It is the case of malvidin-3-*O*-(*p*-coumaroyl)glucoside, which comes from the combination of malvidin-3-*O*-glucoside (which starts to oxidize at +0.55 V) with a coumaric acid molecule (which starts to oxidize at +0.47 V). Malvidin-3-*O*-(*p*-coumaroyl)glucoside is highly represented in the Syrah extract (10.05 mg/g dr in the French extract) but, although its contribution to the antioxidant activity cannot be calculated in the absence of its standard, it is conceivable that it is very low or even null when the redox potentials of the original molecules are considered. 

All the considerations made so far are based on in vitro studies, chemical or electrochemical analyses, but the difference between antioxidant capacity and real antioxidant activity, should not ignore the actual biological activity of the polyphenols. It is known that polyphenols decrease DNA damage induced by various carcinogens acting as ROS scavengers, chelating transition metals, or modulating the expression and the activity of the enzymes related to oxidative stress [8]. As many dietary polyphenols, the grapevine ones have been correlated with a decreased risk of cancer. Some of them, such as resveratrol or epigallocatechin gallate, have been indicated as potential antitumoral alone or in combination with chemotherapeutics [8]. Red wine resveratrol has been known to induce apoptotic cell death in prostate cancer cells [65], and in the HL60 human leukemia cell line, but not in normal human peripheral blood lymphocytes [66]. Vermentino leaf hydroalcoholic extracts lower the cell viability of MCF-7 and SKBR-3 breast cancer cells by a variety of mechanisms [53]. The intake, through a moderate consumption of wine, of resveratrol, quercetin, or the anthocyanins delphinidin and cyanidin, all molecules that we have found in Syrah and Chardonnay extracts, has determined cancer protective effects [67,68]. Epigallocatechin, another molecule we identified in Syrah and Chardonnay pomaces, exerted an anti-invasive effect in ECV304 human endothelial cells by controlling MMP-9 expression through the suppression of ROS, NF-κB, and AP-1 [69]. All the mentioned studies reflect the ability of polyphenols to scavenge endogenously generated oxygen radicals, but some others indicate that antioxidant properties cannot fully account for their chemopreventive effects [8,54].

### 4.3. Polyphenols and Their Prooxidant Properties

It was suggested, in the previous paragraph, that the antioxidant effects of polyphenols could be essential but not sufficient for chemoprevention. There is evidence that naturally occurring antioxidants elicit different redox responses according to a dose-response mechanism and the intracellular redox state [6,7]. The role of ROS as regulators of cellular processes has been widely studied, including in melanoma cancer cells [70]. The malignant phenotype of murine melanoma B16-BL6 cells can be reversed by decreasing the level of ROS using antioxidant enzymes such as SOD [71]; ROS can interact with protein, kinases, and transcription factors through different pathways, leading to the regulation of several processes (proliferation, differentiation, apoptosis) depending on the nature and duration of the stimuli [8]. It was demonstrated that high resveratrol concentration altered the cell redox state of human endothelial cells causing mitochondrial-dependent cell death [72]. Later this concept evolved: high doses of resveratrol reduced the protein kinase C activity, inhibited DNA synthesis, and induced apoptosis of endothelial cells, whereas low resveratrol concentration elicited an opposite effect [73]. The results of this work indicated that the viability of B16F10 melanoma cancer cells was higher than the control when a low to moderate dose of extracts was administered, while it was reduced when high doses were dispensed. So, the hypothesis of a biphasic dose-response took place; low doses of polyphenols exert antioxidant activity promoting cell growth, while higher doses exert prooxidant activity increasing the mortality of B16F10 cells. According to previous studies, it appears as the Syrah and Chardonnay extracts have a hormesis-like behavior [74].

Ours is not the first work that investigated, by CV, the antioxidant and prooxidant activity of pomace extracts obtained from *V. vinifera*. Previous research, based on the assumption that polyphenols with oxidation potential between 0.2 and 0.45 V exert antioxidant activity, while those with oxidation potential greater than 0.45 V exert prooxidant activity [75], tried unsuccessfully to correlate these properties with the inhibition of *Botrytis cinerea* mycelial growth [32]. We agree with those authors on the concept that the antioxidant activity is exerted by the polyphenols which oxidize at low potentials; we agree less with the statement that, on a scale from 0 to 1 V, the polyphenols to the left of the 0.45 V threshold are antioxidants, and those to the right have prooxidant activity. Our biological results lead to other reasoning since the same molecule can act as an antioxidant or prooxidant according to its redox state [73]. Starting from the assumption that most antioxidants of plants origin protect against ROS in some cases and promote radical generation in others, Khan et al. [4] demonstrated that plant polyphenols behave as prooxidants in the presence of copper ions catalyzing DNA breakage through the generation of ROS, and that this breakage correlates with polyphenols apoptotic inducing capacity. Cancer cells are under persistent oxidative stress and have an altered antioxidant defense system; when this stress exceeds a certain threshold, it can lead to apoptosis [76]. Resveratrol, which is a generally effective antioxidant, can switch to prooxidant in the presence of Cu(II) to induce DNA damage [77]. The anthocyanin delphinidin is not only able of binding to DNA and copper, but also catalyzes their redox cycling [78]. It was hypothesized that a redox reaction of polyphenol and Cu(II) in a three-component system DNA-quercetin-Cu(II) may occur, leading to the reduction of Cu(II) to Cu(I), whose re-oxidation generates a variety of ROS; these ROS, in the presence of molecular oxygen, presumably lead to oxidative DNA cleavage [54]. Many of the aforementioned molecules are present in the pomace extracts whose properties are studied in this work. Traces of copper were also found in the extracts, both in Syrah and Chardonnay, presumably residues of phytosanitary treatments with cupric compounds on the grapes. A high correlation between Cu content and prooxidant activity was found in Syrah but not in Chardonnay. Since it has been shown that copper addition increases the susceptibility to undergo apoptosis in rat thymocytes [79], and that copper administration to rats leads to an enhancement of polyphenols-induced DNA breakage in lymphocytes [80], we believe that the copper found in Syrah pomace extracts could play a key role in their prooxidant activity. We do not have enough evidence to assume the same for Chardonnay.

Once established that, and at what concentration, the phenolic extracts of pomaces exert their prooxidant activity, we investigated the ability of 250 μg/mL of extract (the maximum non-toxic dose for fibroblast), to interact with hydrogen peroxide, a stress inducer widely used in biological tests. The data demonstrated that it is possible to obtain a prooxidant effect on cancer cells by combining the extracts with doses of H_2_O_2_ that did not have any toxic effect on fibroblasts, and that the same effect, using only hydrogen peroxide, can be obtained only with cytotoxic doses for the normal cells.

Finally, we investigated the capacity of the extracts to limit the progression of B16F10 cells to leave their primary site. We observed a 40 to 50% reduction, vs. control, in the ability of treated B16F10 to disseminate from the primary tumor site. The results are encouraging but only preliminary, and need further studies to be confirmed.

All these results, in perspective, open further studies on the co-administration of phenolic complexes with clinically standardized therapies. In the past decade, a number of molecules with antitumor activity targeting proteins involved in melanoma pathogenesis [81], with particular reference to BRAF mutant inhibitors (such as dabrafenib or encometinib) administered in combination with MEK inhibitors (trametinib or binimetinib, respectively) are successfully introduced into the clinical practice [82]. Moreover, a new class of immunotherapies, namely the immune checkpoint inhibitors (ICI), is providing a very long-term benefit for patients with advanced or metastatic melanoma [83]. Despite the two types of treatment being used in sequence or combination [84], about 50% of advanced melanoma patients are not responsive or refractory to such treatments, thus opening the field to exploit the use of additional pharmacological compounds. In our case, the here-demonstrated prooxidant effect of the phenolic extracts of pomaces will be firstly tested for their antiproliferative and proapoptotic activity on melanoma cell lines established from patients at various stages of the disease and characterized by different molecular features (BRAF or NRAS mutation, high or low tumor mutation burden, etc.), as already being pursued [85]. We even planned to indeed evaluate the effects of the phenolic extracts of pomaces in inhibiting tumor growth on in vivo mouse models, given alone or in combination with targeted therapies and/or ICI treatments.

## 5. Conclusions

This study showed that Syrah and Chardonnay pomace extracts contained considerable amounts of flavonoids, residues of the extraction during the winemaking process. The extraction made according to a green protocol resulted in the recovery of a high content of anthocyanins and flavan-3-ols, and in ethanol-free extracts, non-toxic and, therefore, suitable for administration to cancerous and healthy cells. The correlations found between the analytical tests and the results of the chemical characterization indicated that the properties of the extracts cannot be defined only by some components, even if much more represented than others, but that synergies and antagonisms between all classes of compounds must be taken into account. Analytical and biological tests have shown that Syrah and Chardonnay pomace extracts have antioxidant or prooxidant activity depending on the time and dose at which they are administered. This dual potential suggests the opportunity to exploit the winemaking by-products for the production of cosmeceuticals, or in combination with clinically standardized anticancer therapies.

## Figures and Tables

**Table 1:** Area under curve of CVs of Syrah and Chardonnay pomace extracts, total polyphenol content measured by Folin–Ciocalteu, and AAox measured by DPPH and CUPRAC.

		ITALY	FRANCE	TUNISIA		ITALY	FRANCE	TUNISIA
	E_app_	AUC (μC)				TPC (mg GAE/g dr)		
**Syrah**	+ 0.8 V	5.93 b	7.35 a	5.05 c	Syrah	232.2 b	299.4 a	217.3 b
**Chardonnay**	+ 0.8 V	5.43 b	6.55 a	6.47 a	Chardonnay	321.8 a	102.9 c	155.9 b
**Syrah**	+ 0.5 V	2.30 b	3.02 a	1.80 c	DPPH (mmol TEAC/g dr)			
					Syrah	1.44 b	1.77 a	1.39 b
					Chardonnay	1.81 a	0.62 c	1.05 b
**Chardonnay**	+ 0.5 V	1.89 b	2.33 a	2.27 a	CUPRAC (mmol Fe^2+^/g dr)			
					Syrah	5.12 b	8.93 a	5.24 b
					Chardonnay	9.78 a	2.82 c	3.52 b

Means in rows followed by unlike letters differ significantly by Fisher’s LSD procedure, *p* ≤ 0.05.

**Table 2:** Contribution of main polyphenols to the total antioxidant activity of pomace extracts.

POLYPHENOLS (Standard)			Contribution of Polyphenols to the Total Antioxidant Activity					
			(Equivalent Millimoles of GA)					
	Redox Potential	Current at +0.5V	SYRAH Extracts			CHARDONNAY Extracts		
	V	μA	France	Italy	Tunisia	France	Italy	Tunisia
								
Anthocyanins								
Peonidin-3-*O*-glucoside	+ 0.23	12.47	2.04	0.44	0.41	-	-	-
Malvidin-3-*O*-glucoside *	+ 0.55	1.22	none	none	none	-	-	-
Flavonols								
								
**Hydroxycinnamic acids**								
								
**Hydroxybenzoic acids**								
Gallic acid	+ 0.18	10.61	3.82	5.70	4.06	1.33	3.15	1.52
**Flavan 3-ols**								
Procyanidin B1	+ 0.16	22.98	3.21	8.68	11.89	1.02	2.49	6.86
(+)-Catechin	+ 0.12	22.96	54.88	30.32	31.03	25.62	70.75	27.93
Procyanidin B2	+ 0.16	21.35	17.79	11.36	8.24	6.72	11.44	8.61
(−)-Epicatechin	+ 0.12	14.26	28.07	18.49	12.76	17.47	40.11	13.78
Epigallocatechin	+ 0.08	19.56	6.66	0.14	0.05	0.19	3.70	0.28

* The contribution of the molecule with a redox potential > 0.5 V has to be considered equal to zero.

**Table 3:** Prooxidant activity of 250 mg/mL of Italian, French, and Tunisian Syrah and Chardonnay pomaces extracts on B16F10 melanoma cancer cells.

		Prooxidant Activity	
**Variety**	**Origin**	Viability (ratio vs. control)	μmoles equivalents of H_2_O_2_
	Italy	0.66 ± 0.05 b	206.00 ± 15.53 b
Syrah	France	0.74 ± 0.03 a	172.96 ± 7.02 c
	Tunisia	0.51 ± 0.03 c	272.96 ± 16.09 a
	Italy	1.08 ± 0.02 a	24.70± 0.46 c
Chardonnay	France	0.94 ± 0.05 b	87.74 ± 4.69 b
	Tunisia	0.83 ± 0.07 c	135.13 ± 11.45 a

Means in column followed by unlike letters differ significantly by Fisher’s LSD procedure, *p* ≤ 0.05. The statistical comparison was made only intra-variety, not inter-varieties.

**Table 4:** Prooxidant activity of combined treatments with 250 μg/mL of Syrah or Chardonnay extract with 50 and 100 μM of H_2_O_2_.

Combined Treatment		Italy	France	Tunisia	Italy	France	Tunisia
250 μg/mL	μM H_2_O_2_	Reduction of Viability (%) vs. Control			μmoles of H_2_O_2_ alone		
Syrah	50	44.72	ne	55.53	253.91	ne	300.90
	100	66.52	76.10	79.25	348.71	390.33	404.03
Chardonnay	50	ne	ne	45.15	ne	ne	255.79
	100	60.65	59.48	64.30	323.19	318.09	339.04

All the means in table differ significantly from the Control (*p* ≤ 0.01). ne=not effective (when the reduction in viability compared to the control is less than 20%).

## Data Availability

Not applicable.

## Supplementary Materials

The following supporting information can be downloaded at: https://www.mdpi.com/article/10.3390/antiox12010080/s1, Figure S1: HPLC-DAD chromatograms of Italian Syrah (A) and Tunisian Chardonnay (B) pomaces extracts; Figure S2: Gallic acid calibration curve; Figure S3. Effects of 24 h treatments on fibroblasts; Figure S4. Dose-response test with increasing quantity of H_2_O_2_ on B16F10 cells (Figure S4A) and fibroblasts (Figure S4B), and H_2_O_2_ calibration curve (Figure S4C); Table S1: Targeted phenolic compounds of analysed Italian, French and Tunisian Syrah pomace extracts (mg/g dr); Table S2: Targeted phenolic compounds of analysed Italian, French, and Tunisian Syrah pomace extracts (mg/g dr).

## References

[1] Asensi M., Ortega A., Mena S., Feddi F., Estrela J.M. (2011). Natural Polyphenols in Cancer Therapy. Crit. Rev. Clin. Lab. Sci..

[2] Yammine A., Namsi A., Vervandier-Fasseur D., Mackrill J.J., Lizard G., Latruffe N. (2021). Polyphenols of the Mediterranean Diet and Their Metabolites in the Prevention of Colorectal Cancer. Molecules.

[3] Sotler R., Poljšak B., Dahmane R., Jukić T., Pavan Jukić D., Rotim C., Trebše P., Starsc A. (2019). Prooxidant activities of antioxidants and their impact on health. Acta Clin. Croat..

[4] Khan H.Y., Hadi S.M., Mohammad R.M., Azmi A.S., Kabir Y. (2020). 12—Prooxidant Anticancer Activity of Plant-Derived Polyphenolic Compounds: An Underappreciated Phenomenon. Functional Foods in Cancer Prevention and Therapy.

[5] Yang L., Jia L., Li X., Zhang K., Wang X., He Y., Hao M., Rayman M.P., Zhang J. (2022). Prooxidant Activity-Based Guideline for a Beneficial Combination of (−)-Epigallocatechin-3-Gallate and Chlorogenic Acid. Food Chem..

[6] Pasciu V., Posadino A.M., Cossu A., Sanna B., Tadolini B., Gaspa L., Marchisio A., Dessole S., Capobianco G., Pintus G. (2010). Akt Downregulation by Flavin Oxidase–Induced ROS Generation Mediates Dose-Dependent Endothelial Cell Damage Elicited by Natural Antioxidants. Toxicol. Sci..

[7] Giordo R., Cossu A., Pasciu V., Hoa P.T., Posadino A.M., Pintus G. (2013). Different Redox Response Elicited by Naturally Occurring Antioxidants in Human Endothelial Cells. Open Biochem. J..

[8] León-González A.J., Auger C., Schini-Kerth V.B. (2015). Pro-Oxidant Activity of Polyphenols and Its Implication on Cancer Chemoprevention and Chemotherapy. Biochem. Pharmacol..

[9] Radin N.S. (2003). Designing Anticancer Drugs via the Achilles Heel: Ceramide, Allylic Ketones, and Mitochondria. Bioorganic Med. Chem..

[10] Kang K.A., Piao M.J., Hyun Y.J., Zhen A.X., Cho S.J., Ahn M.J., Yi J.M., Hyun J.W. (2019). Luteolin Promotes Apoptotic Cell Death via Upregulation of Nrf2 Expression by DNA Demethylase and the Interaction of Nrf2 with P53 in Human Colon Cancer Cells. Exp. Mol. Med..

[11] Das L., Vinayak M. (2015). Long Term Effect of Curcumin in Restoration of Tumour Suppressor P53 and Phase-II Antioxidant Enzymes via Activation of Nrf2 Signalling and Modulation of Inflammation in Prevention of Cancer. PLoS ONE.

[12] Gomez-Cadena A., Urueña C., Prieto K., Martinez-Usatorre A., Donda A., Barreto A., Romero P., Fiorentino S. (2016). Immune-System-Dependent Anti-Tumor Activity of a Plant-Derived Polyphenol Rich Fraction in a Melanoma Mouse Model. Cell Death Dis..

[13] Heenatigala Palliyage G., Singh S., Ashby C.R., Tiwari A.K., Chauhan H. (2019). Pharmaceutical Topical Delivery of Poorly Soluble Polyphenols: Potential Role in Prevention and Treatment of Melanoma. AAPS PharmSciTech.

[14] AlQathama A., Prieto J.M. (2015). Natural Products with Therapeutic Potential in Melanoma Metastasis. Nat. Prod. Rep..

[15] Yen G.-C., Chen H.-Y., Peng H.-H. (1997). Antioxidant and Pro-Oxidant Effects of Various Tea Extracts. J. Agric. Food Chem..

[16] Hadi S.M., Asad S.F., Singh S., Ahmad A. (2000). Putative Mechanism for Anticancer and Apoptosis-Inducing Properties of Plant-Derived Polyphenolic Compounds. IUBMB Life.

[17] Hadi S.M., Bhat S.H., Azmi A.S., Hanif S., Shamim U., Ullah M.F. (2007). Oxidative Breakage of Cellular DNA by Plant Polyphenols: A Putative Mechanism for Anticancer Properties. Semin. Cancer Biol..

[18] Sadowska-Bartosz I., Bartosz G. (2022). Evaluation of The Antioxidant Capacity of Food Products: Methods, Applications and Limitations. Processes.

[19] Munteanu I.G., Apetrei C. (2022). A Review on Electrochemical Sensors and Biosensors Used in Assessing Antioxidant Activity. Antioxidants.

[20] Alam M.W., Najeeb J., Naeem S., Usman S.M., Nahvi I., Alismail F., Abuzir A., Farhan M., Nawaz A. (2022). Electrochemical Methodologies for Investigating the Antioxidant Potential of Plant and Fruit Extracts: A Review. Antioxidants.

[21] Zheng Y., Karimi-Maleh H., Fu L. (2022). Evaluation of Antioxidants Using Electrochemical Sensors: A Bibliometric Analysis. Sensors.

[22] Haque M.A., Morozova K., Ferrentino G., Scampicchio M. (2021). Electrochemical Methods to Evaluate the Antioxidant Activity and Capacity of Foods: A Review. Electroanalysis.

[23] Percevault L., Limanton E., Nicolas P., Paquin L., Lagrost C. (2021). Electrochemical Determination and Antioxidant Capacity Modulation of Polyphenols in Deep Eutectic Solvents. ACS Sustain. Chem. Eng..

[24] Karadag A., Ozcelik B., Saner S. (2009). Review of Methods to Determine Antioxidant Capacities. Food Anal. Methods.

[25] Intarakamhang S., Leson C., Schuhmann W., Schulte A. (2011). A Novel Automated Electrochemical Ascorbic Acid Assay in the 24-Well Microtiter Plate Format. Anal. Chim. Acta.

[26] Buratti S., Scampicchio M., Giovanelli G., Mannino S. (2008). A Low-Cost and Low-Tech Electrochemical Flow System for the Evaluation of Total Phenolic Content and Antioxidant Power of Tea Infusions. Talanta.

[27] Spissu Y., Barberis A., D’hallewin G., Orrù G., Scano A., Serra G.R., Pinna M., Pinna C., Marceddu S., Serra P.A. (2021). An Ascorbate Bluetooth© Analyzer for Quality Control of Fresh-Cut Parsley Supply Chain. Antioxidants.

[28] Spissu Y., Barberis A., Bazzu G., D’hallewin G., Rocchitta G., Serra P.A., Marceddu S., Vineis C., Garroni S., Culeddu N. (2021). Functionalization of Screen-Printed Sensors with a High Reactivity Carbonaceous Material for Ascorbic Acid Detection in Fresh-Cut Fruit with Low Vitamin C Content. Chemosensors.

[29] Pedotti S., Patti A., Dedola S., Barberis A., Fabbri D., Dettori M.A., Serra P.A., Delogu G. (2016). Synthesis of New Ferrocenyl Dehydrozingerone Derivatives and Their Effects on Viability of PC12 Cells. Polyhedron.

[30] Sun-Waterhouse D., Smith B.G., O’Connor C.J., Melton L.D. (2008). Effect of Raw and Cooked Onion Dietary Fibre on the Antioxidant Activity of Ascorbic Acid and Quercetin. Food Chem..

[31] Piljac-Žegarac J., Valek L., Stipčević T., Martinez S. (2010). Electrochemical Determination of Antioxidant Capacity of Fruit Tea Infusions. Food Chem..

[32] Cotoras M., Vivanco H., Melo R., Aguirre M., Silva E., Mendoza L. (2014). In Vitro and In Vivo Evaluation of the Antioxidant and Prooxidant Activity of Phenolic Compounds Obtained from Grape (*Vitis vinifera*) Pomace. Molecules.

[33] Zielinska D., Wiczkowski W., Piskula M.K. (2008). Determination of the Relative Contribution of Quercetin and Its Glucosides to the Antioxidant Capacity of Onion by Cyclic Voltammetry and Spectrophotometric Methods. J. Agric. Food Chem..

[34] Makhotkina O., Kilmartin P.A. (2010). The Use of Cyclic Voltammetry for Wine Analysis: Determination of Polyphenols and Free Sulfur Dioxide. Anal. Chim. Acta.

[35] Milia E., Usai M., Szotáková B., Elstnerová M., Králová V., D’hallewin G., Spissu Y., Barberis A., Marchetti M., Bortone A. (2020). The Pharmaceutical Ability of *Pistacia lentiscus* L. Leaves Essential Oil against Periodontal Bacteria and *Candida* sp. and Its Anti-Inflammatory Potential. Antibiotics.

[36] Barberis A., Deiana M., Spissu Y., Azara E., Fadda A., Serra P.A., D’hallewin G., Pisano M., Serreli G., Orrù G. (2020). Antioxidant, Antimicrobial, and Other Biological Properties of Pompia Juice. Molecules.

[37] Russo G.L., Russo M., Spagnuolo C., Tedesco I., Bilotto S., Iannitti R., Palumbo R., Zappia V., Panico S., Russo G.L., Budillon A., Della Ragione F. (2014). Quercetin: A Pleiotropic Kinase Inhibitor against Cancer BT—Advances in Nutrition and Cancer.

[38] Antonić B., Jančíková S., Dordević D., Tremlová B. (2020). Grape Pomace Valorization: A Systematic Review and Meta-Analysis. Foods.

[39] Lu Y., Foo L.Y. (1999). The Polyphenol Constituents of Grape Pomace. Food Chem..

[40] García-Lomillo J., González-SanJosé M.L. (2017). Applications of Wine Pomace in the Food Industry: Approaches and Functions. Compr. Rev. Food Sci. Food Saf..

[41] Chedea V.S., Braicu C., Socaciu C. (2010). Antioxidant/Prooxidant Activity of a Polyphenolic Grape Seed Extract. Food Chem..

[42] Beres C., Costa G.N.S., Cabezudo I., da Silva-James N.K., Teles A.S.C., Cruz A.P.G., Mellinger-Silva C., Tonon R.V., Cabral L.M.C., Freitas S.P. (2017). Towards Integral Utilization of Grape Pomace from Winemaking Process: A Review. Waste Manag..

[43] Rockenbach I.I., Jungfer E., Ritter C., Santiago-Schübel B., Thiele B., Fett R., Galensa R. (2012). Characterization of Flavan-3-Ols in Seeds of Grape Pomace by CE, HPLC-DAD-MSn and LC-ESI-FTICR-MS. Food Res. Int..

[44] Spigno G., Tramelli L., De Faveri D.M. (2007). Effects of Extraction Time, Temperature and Solvent on Concentration and Antioxidant Activity of Grape Marc Phenolics. J. Food Eng..

[45] José Jara-Palacios M., Hernanz D., Luisa Escudero-Gilete M., Heredia F.J. (2014). Antioxidant Potential of White Grape Pomaces: Phenolic Composition and Antioxidant Capacity Measured by Spectrophotometric and Cyclic Voltammetry Methods. Food Res. Int..

[46] BestMedGrape Project. https://www.enicbcmed.eu/projects/bestmedgrape.

[47] Fadda A., Pace B., Angioni A., Barberis A., Cefola M. (2016). Suitability for Ready-to-Eat Processing and Preservation of Six Green and Red Baby Leaves Cultivars and Evaluation of Their Antioxidant Value during Storage and after the Expiration Date. J. Food Process. Preserv..

[48] Perra M., Cuena-Lombraña A., Bacchetta G., Manca M.L., Manconi M., Maroun R.G., Muntoni A., Tuberoso C.I., Gil K.A., De Gioannis G. (2022). Combining Different Approaches for Grape Pomace Valorization: Polyphenols Extraction and Composting of the Exhausted Biomass. Sustainability.

[49] Barberis A., Spissu Y., Bazzu G., Fadda A., Azara E., Sanna D., Schirra M., Serra P.A. (2014). Development and Characterization of an Ascorbate Oxidase-Based Sensor–Biosensor System for Telemetric Detection of AA and Antioxidant Capacity in Fresh Orange Juice. Anal. Chem..

[50] Barberis A., Spissu Y., Fadda A., Azara E., Bazzu G., Marceddu S., Angioni A., Sanna D., Schirra M., Serra P.A. (2015). Simultaneous Amperometric Detection of Ascorbic Acid and Antioxidant Capacity in Orange, Blueberry and Kiwi Juice, by a Telemetric System Coupled with a Fullerene- or Nanotubes-Modified Ascorbate Subtractive Biosensor. Biosens. Bioelectron..

[51] Bouzabata A., Montoro P., Gil K.A., Piacente S., Youssef F.S., Al Musayeib N.M., Cordell G.A., Ashour M.L., Tuberoso C.I.G. (2022). HR-LC-ESI-Orbitrap-MS-Based Metabolic Profiling Coupled with Chemometrics for the Discrimination of Different Echinops Spinosus Organs and Evaluation of Their Antioxidant Activity. Antioxidants.

[52] Tuberoso C.I.G., Serreli G., Congiu F., Montoro P., Fenu M.A. (2017). Characterization, Phenolic Profile, Nitrogen Compounds and Antioxidant Activity of Carignano Wines. J. Food Compos. Anal..

[53] Floris A., Mazarei M., Yang X., Robinson A.E., Zhou J., Barberis A., D’hallewin G., Azara E., Spissu Y., Iglesias-Ara A. (2020). SUMOylation Protects FASN against Proteasomal Degradation in Breast Cancer Cells Treated with Grape Leaf Extract. Biomolecules.

[54] Khan H.Y., Zubair H., Ullah M.F., Ahmad A., Hadi S.M. (2012). A Prooxidant Mechanism for the Anticancer and Chemopreventive Properties of Plant Polyphenols. Curr. Drug Targets.

[55] Pijuan J., Barceló C., Moreno D.F., Maiques O., Sisó P., Marti R.M., Macià A., Panosa A. (2019). In Vitro Cell Migration, Invasion, and Adhesion Assays: From Cell Imaging to Data Analysis. Front. Cell Dev. Biol..

[56] Ky I., Teissedre P.-L. (2015). Characterisation of Mediterranean Grape Pomace Seed and Skin Extracts: Polyphenolic Content and Antioxidant Activity. Molecules.

[57] Alonso Á.M., Guillén D.A., Barroso C.G., Puertas B., García A. (2002). Determination of Antioxidant Activity of Wine Byproducts and Its Correlation with Polyphenolic Content. J. Agric. Food Chem..

[58] Lingua M.S., Fabani M.P., Wunderlin D.A., Baroni M.V. (2016). From Grape to Wine: Changes in Phenolic Composition and Its Influence on Antioxidant Activity. Food Chem..

[59] de la Cerda-Carrasco A., López-Solís R., Nuñez-Kalasic H., Peña-Neira Á., Obreque-Slier E. (2015). Phenolic Composition and Antioxidant Capacity of Pomaces from Four Grape Varieties (*Vitis vinifera* L.). J. Sci. Food Agric..

[60] Hollecker L., Pinna M., Filippino G., Scrugli S., Pinna B., Argiolas F., Murru M. (2009). Simultaneous Determination of Polyphenolic Compounds in Red and White Grapes Grown in Sardinia by High Performance Liquid Chromatography–Electron Spray Ionisation-Mass Spectrometry. J. Chromatogr. A.

[61] Rodríguez Montealegre R., Romero Peces R., Chacón Vozmediano J.L., Martínez Gascueña J., García Romero E. (2006). Phenolic Compounds in Skins and Seeds of Ten Grape Vitis Vinifera Varieties Grown in a Warm Climate. J. Food Compos. Anal..

[62] Makhotkina O., Kilmartin P.A. (2009). Uncovering the Influence of Antioxidants on Polyphenol Oxidation in Wines Using an Electrochemical Method: Cyclic Voltammetry. J. Electroanal. Chem..

[63] Kilmartin P.A. (2016). Electrochemistry Applied to the Analysis of Wine: A Mini-Review. Electrochem. Commun..

[64] Piljac J., Martinez S., Stipèeviæ T., Petroviæ E., Metikoš-Hukoviæ M. (2004). Cyclic Voltammetry Investigation of the Phenolic Content of Croatian Wines. Am. J. Enol. Vitic..

[65] Chang K.-L., Cheng H.-L., Huang L.-W., Hsieh B.-S., Hu Y.-C., Chih T.-T., Shyu H.-W., Su S.-J. (2009). Combined Effects of Terazosin and Genistein on a Metastatic, Hormone-Independent Human Prostate Cancer Cell Line. Cancer Lett..

[66] Clément M.-V., Hirpara J.L., Chawdhury S.-H., Pervaiz S. (1998). Chemopreventive Agent Resveratrol, a Natural Product Derived From Grapes, Triggers CD95 Signaling-Dependent Apoptosis in Human Tumor Cells. Blood.

[67] He S., Sun C., Pan Y. (2008). Red Wine Polyphenols for Cancer Prevention. Int. J. Mol. Sci..

[68] Levi F., Pasche C., Lucchini F., Ghidoni R., Ferraroni M., La Vecchia C. (2005). Resveratrol and Breast Cancer Risk. Eur. J. Cancer Prev..

[69] Khoi Ngoc P., Park Sun J., Kim Hee J., Xia Y., Kim Ho N., Kim Keun K., Jung Do Y. (2013). (−)-Epigallocatechin-3-Gallate Blocks Nicotine-Induced Matrix Metalloproteinase-9 Expression and Invasiveness via Suppression of NF-ΚB and AP-1 in Endothelial Cells. Int. J. Oncol..

[70] Venza I., Venza M., Visalli M., Lentini G., Teti D., D’Alcontres F.S. (2021). ROS as Regulators of Cellular Processes in Melanoma. Oxidative Med. Cell. Longev..

[71] Hyoudou K., Nishikawa M., Umeyama Y., Kobayashi Y., Yamashita F., Hashida M. (2004). Inhibition of Metastatic Tumor Growth in Mouse Lung by Repeated Administration of Polyethylene Glycol-Conjugated Catalase: Quantitative Analysis with Firefly Luciferase-Expressing Melanoma Cells. Clin. Cancer Res..

[72] Posadino A.M., Cossu A., Giordo R., Zinellu A., Sotgia S., Vardeu A., Hoa P.T., Nguyen L.H.V., Carru C., Pintus G. (2015). Resveratrol Alters Human Endothelial Cells Redox State and Causes Mitochondrial-Dependent Cell Death. Food Chem. Toxicol..

[73] Posadino A.M., Giordo R., Cossu A., Nasrallah G.K., Shaito A., Abou-Saleh H., Eid A.H., Pintus G. (2019). Flavin Oxidase-Induced ROS Generation Modulates PKC Biphasic Effect of Resveratrol on Endothelial Cell Survival. Biomolecules.

[74] Calabrese E.J., Mattson M.P., Dhawan G., Kapoor R., Calabrese V., Giordano J., Söderbom G., Esterline R., Oscarsson J., Mattson M. (2020). Chapter Ten—Hormesis: A Potential Strategic Approach to the Treatment of Neurodegenerative Disease. Metabolic and Bioenergetic Drivers of Neurodegenerative Disease: Treating Neurodegenerative Diseases as Metabolic Diseases.

[75] Simić A., Manojlović D., Šegan D., Todorović M. (2007). Electrochemical Behavior and Antioxidant and Prooxidant Activity of Natural Phenolics. Molecules.

[76] Gupte A., Mumper R.J. (2009). Elevated Copper and Oxidative Stress in Cancer Cells as a Target for Cancer Treatment. Cancer Treat. Rev..

[77] Zheng L.-F., Wei Q.-Y., Cai Y.-J., Fang J.-G., Zhou B., Yang L., Liu Z.-L. (2006). DNA Damage Induced by Resveratrol and Its Synthetic Analogues in the Presence of Cu (II) Ions: Mechanism and Structure-Activity Relationship. Free Radic. Biol. Med..

[78] Hanif S., Shamim U., Ullah M.F., Azmi A.S., Bhat S.H., Hadi S.M. (2008). The Anthocyanidin Delphinidin Mobilizes Endogenous Copper Ions from Human Lymphocytes Leading to Oxidative Degradation of Cellular DNA. Toxicology.

[79] Wolfe J.T., Ross D., Cohen G.M. (1994). A Role for Metals and Free Radicals in the Induction of Apoptosis in Thymocytes. FEBS Lett..

[80] Khan H.Y., Zubair H., Ullah M.F., Ahmad A., Hadi S.M. (2011). Oral Administration of Copper to Rats Leads to Increased Lymphocyte Cellular DNA Degradation by Dietary Polyphenols: Implications for a Cancer Preventive Mechanism. BioMetals.

[81] Palmieri G., Colombino M., Casula M., Manca A., Mandalà M., Cossu A., Antonio Cossu for the Italian Melanoma Intergroup (IMI) (2018). Molecular Pathways in Melanomagenesis: What We Learned from Next-Generation Sequencing Approaches. Curr. Oncol. Rep..

[82] Crispo A., Corradin M.T., Giulioni E., Vecchiato A., Del Fiore P., Queirolo P., Spagnolo F., Vanella V., Caracò C., Tosti G. (2021). Real Life Clinical Management and Survival in Advanced Cutaneous Melanoma: The Italian Clinical National Melanoma Registry Experience. Front. Oncol..

[83] Palmieri G., Puzanov I., Massi D., Ascierto P.A. (2021). Editorial: Advancements in Molecular Diagnosis and Treatment of Melanoma. Front. Oncol..

[84] Ascierto P.A., Mandalà M., Ferrucci P.F., Guidoboni M., Rutkowski P., Ferraresi V., Arance A., Guida M., Maiello E., Gogas H. (2022). Sequencing of Ipilimumab Plus Nivolumab and Encorafenib Plus Binimetinib for Untreated BRAF-Mutated Metastatic Melanoma (SECOMBIT): A Randomized, Three-Arm, Open-Label Phase II Trial. J. Clin. Oncol..

[85] Pisano M., Dettori M.A., Fabbri D., Delogu G., Palmieri G., Rozzo C. (2021). Anticancer Activity of Two Novel Hydroxylated Biphenyl Compounds toward Malignant Melanoma Cells. Int. J. Mol. Sci..

