# Supplementary material for: Anti- and Pro-Oxidant Activity of Polyphenols Extracts of Syrah and Chardonnay Grapevine Pomaces on Melanoma Cancer Cells
Source: Antioxidants (Basel). 2022 Dec 29;12(1):80. doi: 10.3390/antiox12010080 (PMC9855015; doi:10.3390/antiox12010080)
Supplement: Supplementary file 1 [file antioxidants-12-00080-s001.zip › antioxidants-2113156-supplementary.pdf]

ANTI- AND PRO-OXIDANT ACTIVITY OF POLYPHENOLS EXTRACTS OF SYRAH AND CHARDONNAY GRAPEVINE POMACES ON MELANOMA CANCER CELLS

Ylenia Spissu [1], Katarzyna Angelika Gil [2], Antonio Dore [1], Giulia Sanna [1], Giuseppe Palmieri [3], Andrea Sanna [4], Maurizio Cossu [4], Feten Belhadj [5], Boutheina Gharbi [5], Maria Barbara Pinna [6], Antonio Barberis [1*], Carlo Ignazio Giovanni Tuberoso [2*], and Guy D'hallewin [1]

Supplementary materials

Table S1 - Targeted phenolic compounds of analysed Italian, French and Tunisian Syrah pomace extracts (mg/g dr).

Table S2 - Targeted phenolic compounds of analysed Italian, French and Tunisian Syrah pomace extracts (mg/g dr).

Figure S1. HPLC-DAD chromatograms of Italian Syrah (A) and Tunisian Chardonnay (B) pomaces extracts at λ = 280 nm (for peaks attribution see Table S1 and Table S2). Chromatographic conditions are described in the text.

Figure S2. Gallic acid calibration curve

Figure S3. Effects of 24h treatments on fibroblasts

Figure S4. Dose-response test with increasing quantity of H_2O_2 on B16F10 cells (Figure S4A) and fibroblasts (Figure S4B), and H_2O_2 calibration curve (Figure S4C)

Table S1. Targeted phenolic compounds of analysed Italian, French and Tunisian Syrah pomace extracts (mg/g dr).

Compound mg/g dr	**Id**[a]	N° Fig. S1	France		Italy		Tunisia		Total				
			mean	±SD	mean	±SD	mean	±SD	mean	±SD	median	min	max
**Anthocyanins**													
Delphinidin-3-*O*-glucoside	UV-Vis, tr	10	0.19	0.00	0.07	0.00	0.09	0.00	0.12	0.07	0.09	0.07	0.19
Cyanidin-3-*O*-glucoside	UV-Vis, tr	12	0.04	0.00	0.03	0.00	0.02	0.00	0.03	0.01	0.03	0.02	0.04
Petunidin-3-*O*-glucoside	UV-Vis, tr	14	0.48	0.00	0.15	0.01	0.15	0.00	0.26	0.19	0.15	0.15	0.48
Peonidin-3-*O*-glucoside	UV-Vis, tr	15	0.75	0.00	0.16	0.00	0.15	0.00	0.35	0.35	0.16	0.15	0.75
Malvidin-3-*O*-glucoside	UV-Vis, tr	16	6.43	0.00	1.75	0.06	1.11	0.01	3.09	2.91	1.75	1.11	6.43
Delphinidin-3-(acethyl)-glucoside	UV-Vis	17	0.11	0.02	0.08	0.00	0.07	0.00	0.09	0.02	0.08	0.07	0.11
Peonidin-3-(acethyl)-glucoside	UV-Vis	22	0.30	0.01	0.06	0.00	0.07	0.00	0.14	0.13	0.07	0.06	0.30
Malvidin-3-(acethyl)-glucoside	UV-Vis	24	2.09	0.02	0.83	0.04	0.54	0.03	1.15	0.82	0.83	0.54	2.09
Cyanidin-3-*O*-(*p*-coumaroyl)glucoside	UV-Vis	25	0.41	0.00	0.30	0.00	0.14	0.00	0.29	0.14	0.30	0.14	0.41
Malvidin-3-(caffeoyl)glucoside	UV-Vis	26	0.85	0.02	0.26	0.01	0.08	0.00	0.40	0.40	0.26	0.08	0.85
Petunidin-3-(coumaroyl)glucoside	UV-Vis	27	0.76	0.04	0.60	0.00	0.24	0.00	0.53	0.27	0.60	0.24	0.76
Peonidin-3-*O*-(*p*-coumaroyl)glucoside	UV-Vis	28	1.48	0.02	0.99	0.02	0.34	0.00	0.94	0.57	0.99	0.34	1.48
Malvidin-3-*O*-(*p*-coumaroyl)glucoside	UV-Vis	29	10.05	0.26	7.68	0.13	2.49	0.03	6.74	3.87	7.68	2.49	10.05
Other Anthocyanins[b]	UV-Vis		0.83	0.00	0.60	0.00	0.47	0.00	0.63	0.18	0.60	0.47	0.83
Total Anthocyanins			24.77	0.40	13.55	0.28	5.96	0.09	14.76	9.46	13.55	5.96	24.77
**Flavonols**													
Quercetin-3-*O*-galactoside	UV-Vis, tr	19	0.29	0.01	0.11	0.01	0.71	0.00	0.37	0.31	0.29	0.11	0.71
Quercetin-3-*O*-glucoside	UV-Vis, tr	20	0.17	0.00	0.08	0.00	0.13	0.00	0.13	0.04	0.13	0.08	0.17
Quercetin-3-*O*-glucuronide	UV-Vis, tr	21	0.56	0.00	0.47	0.01	1.17	0.01	0.73	0.38	0.56	0.47	1.17
Kaempferol-3-*O*-glucoside	UV-Vis, tr	23	nd		nd		nd		nd		nd	nd	nd
Quercetin	UV-Vis, tr	30	tr		0.65	0.00	0.33	0.00	0.49	0.22	0.49	0.33	0.65
Kaempferol	UV-Vis, tr	31	tr		0.06	0.00	0.06	0.00	0.06	0.00	0.06	0.06	0.06
Isorhamnetin	UV-Vis, tr	32	tr		0.16	0.00	0.06	0.00	0.11	0.07	0.11	0.06	0.16
Other flavonols[c]	UV-Vis		0.78	0.01	0.64	0.02	0.99	0.01	0.80	0.18	0.78	0.64	0.99
Total Flavonols			1.79	0.02	2.17	0.04	3.44	0.03	2.47	0.86	2.17	1.79	3.44
**Hydroxycinnamic acids**													
Caftaric acid	UV-Vis, tr	3	0.02	0.00	0.22	0.01	0.54	0.02	0.26	0.26	0.22	0.02	0.54
p-Coumaric acid	UV-Vis, tr	7	tr		0.11	0.01	0.07	0.00	0.09	0.03	0.09	0.07	0.11
Other hydroxycinnamic acids[d]	UV-Vis		0.81	0.03	0.83	0.04	0.87	0.01	0.84	0.03	0.83	0.81	0.87
Total hydroxycinnamic acids			0.83	0.03	1.17	0.06	1.48	0.03	1.16	0.33	1.17	0.83	1.48
**Hydroxybenzoic acids**													
Gallic acid	UV-Vis, tr	1	0.63	0.03	0.94	0.03	0.67	0.01	0.75	0.17	0.67	0.63	0.94

Protocatechuic acid	UV-Vis, tr	2	tr		0.12	0.00	0.05	0.00	0.08	0.04	0.08	0.05	0.12
Vanillic acid	UV-Vis, tr	4	0.21	0.02	0.04	0.02	0.04	0.00	0.10	0.10	0.04	0.04	0.21
Syringic acid	UV-Vis, tr	8	0.53	0.00	0.42	0.00	0.16	0.01	0.37	0.19	0.42	0.16	0.53
Ellagic acid	UV-Vis, tr	18	tr		0.05	0.01	0.04	0.00	0.04	0.01	0.04	0.04	0.05
Other hydroxybenzoic acids[e]	UV-Vis		0.17	0.00	0.51	0.04	0.61	0.01	0.43	0.23	0.51	0.17	0.61
Total Hydroxybenzoic acids			1.54	0.05	2.08	0.10	1.56	0.03	1.73	0.30	1.56	1.54	2.08
**Flavan 3-ols**													
Procyanidin B1	UV-Vis, tr	5	0.72	0.07	1.95	0.41	2.67	0.23	1.78	0.99	1.95	0.72	2.67
(+)-Catechin	UV-Vis, tr	6	6.19	0.35	3.42	0.28	3.50	0.01	4.37	1.58	3.50	3.42	6.19
Procyanidin B2	UV-Vis, tr	9	4.34	0.20	2.77	0.42	2.01	0.15	3.04	1.19	2.77	2.01	4.34
(-)-Epicatechin	UV-Vis, tr	11	5.48	0.16	3.61	0.11	2.49	0.14	3.86	1.51	3.61	2.49	5.48
Epigallocatechin	UV-Vis, tr	13	1.42	0.11	0.03	0.00	0.01	0.00	0.49	0.81	0.03	0.01	1.42
Other Flavan-3-ols[f]	UV-Vis		13.81	0.03	13.77	0.03	13.79	0.02	13.79	0.02	13.79	13.77	13.81
Total Flavan 3-ols			31.97	0.93	25.55	1.26	24.47	0.55	27.33	4.05	25.55	24.47	31.97
**Total phenolic compounds**			60.90	1.44	44.50	1.75	36.92	0.73	47.44	12.26	44.50	36.92	60.90

[a] Id: identification: Rt, comparison with retention time and UV-VIS spectra of pure standard; UV-Vis, comparison with UV-VIS spectra of pure compound or similar pure standards and literature data; [b]: dosed with the calibration curve of malvidin-3-*O*-glucoside; [c]: dosed with the calibration curve of quercetin-3-*O*-glucoside; [d] dosed with the calibration curve of caftaric acid; [e] dosed with the calibration curve of gallic acid; [f] dosed with the calibration curve of procyanidin B1. The results are reported as mean value ± standard deviation (n = 3).

Table S2. Targeted phenolic compounds of analysed French, Italian and Tunisian Chardonnay pomace extracts (mg/g dr).

Compound mg/g dr	Id[a]	N° Fig. S1	France		Italy		Tunisia		Total				
			mean	±SD	mean	±SD	mean	±SD	mean	±SD	median	min	max
**Anthocyanins**													
Total Anthocyanins			nd		nd		nd		nd		nd		nd
**Flavonols**													
Quercetin-3-*O*-galactoside	UV-Vis, tr	19	1.06	0.00	0.07	0.00	0.39	0.01	0.50	0.51	0.39	0.07	1.06
Quercetin-3-*O*-glucoside	UV-Vis, tr	20	tr		tr		tr		tr		tr	tr	tr
Quercetin-3-*O*-glucuronide	UV-Vis, tr	21	0.46	0.00	0.42	0.00	1.10	0.01	0.66	0.39	0.46	0.42	1.10
Kaempferolo-3-*O*-glucoside	UV-Vis, tr	23	0.42	0.00	tr		0.20	0.00	0.31	0.16	0.31	0.20	0.42
Quercetin	UV-Vis, tr	30	tr		tr		0.12	0.00	0.12	0.00	0.12	0.12	0.12
Kaempferol	UV-Vis, tr	31	tr		tr		0.08	0.01	0.08	0.01	0.08	0.08	0.08
Isorhamnetin	UV-Vis, tr	32	nd		nd		nd		nd		nd	nd	nd
Other flavonols[b]	UV-Vis		0.12	0.00	0.06	0.00	0.15	0.01	0.11	0.04	0.12	0.06	0.15
Total Flavonols			2.05	0.01	0.54	0.01	2.03	0.11	1.54	0.86	2.03	0.54	2.05
**Hydroxycinnamic acids**													
Caftaric acid	UV-Vis, tr	3	0.03	0.00	0.03	0.00	0.16	0.00	0.07	0.08	0.03	0.03	0.16
p-Coumaric acid	UV-Vis, tr	7	tr		tr		0.02	0.00	0.02	0.00	0.02	0.02	0.02
Other hydroxycinnamic acids[c]	UV-Vis		0.34	0.02	0.36	0.01	0.12	0.00	0.27	0.14	0.34	0.12	0.36
Total hydroxycinnamic acids			0.36	0.02	0.40	0.01	0.30	0.01	0.35	0.05	0.36	0.30	0.40
**Hydroxybenzoic acids**													
Gallic acid	UV-Vis, tr	1	0.22	0.01	0.52	0.05	0.25	0.00	0.33	0.16	0.25	0.22	0.52
Protocatechuic acid	UV-Vis, tr	2	tr		tr		0.04	0.00	0.04	0.00	0.04	nd	0.04
Vanillic acid	UV-Vis, tr	4	nd		0.05	0.00	nd		0.05	0.00	0.05	0.05	0.05
Syringic acid	UV-Vis, tr	8	nd		nd		nd		nd	nd	nd	nd	nd
Ellagic acid	UV-Vis, tr	18	nd		tr		0.03	0.00	0.03	0.00	0.03	nd	0.03
Other hydroxybenzoic acids[d]	UV-Vis		0.39	0.02	0.76	0.04	0.32	0.00	0.49	0.23	0.39	0.32	0.76
Total Hydroxybenzoic acids			0.61	0.02	1.32	0.10	0.64	0.01	0.86	0.40	0.64	0.61	1.32
**Flavan 3-ols**													
Procyanidin B1	UV-Vis, tr	5	0.23	0.02	0.56	0.14	1.54	0.08	0.77	0.68	0.56	0.23	1.54
(+)-Catechin	UV-Vis, tr	6	2.89	0.23	7.98	1.08	3.15	0.04	4.67	2.86	3.15	2.89	7.98
Procyanidin B2	UV-Vis, tr	9	1.64	0.12	2.79	0.67	2.10	0.07	2.18	0.58	2.10	1.64	2.79
(-)-Epicatechin	UV-Vis, tr	11	3.41	0.18	7.83	0.02	2.69	0.02	4.64	2.78	3.41	2.69	7.83
Epigallocatechin	UV-Vis, tr	13	0.04	0.00	0.79	0.15	0.06	0.01	0.30	0.43	0.06	0.04	0.79
Other Flavan-3-ols[e]	UV-Vis		7.05	0.02	12.23	0.06	10.10	0.01	9.79	2.60	10.10	7.05	12.23
Total Flavan 3-ols			15.26	0.57	32.18	2.13	19.64	0.22	22.36	8.78	19.64	15.26	32.18

**Total phenolic compounds**			18.28	0.61	34.44	2.25	22.62	0.35	25.11	8.36	22.62	18.28	34.44

[a] Id: identification: Rt, comparison with retention time and UV-VIS spectra of pure standard; UV-Vis, comparison with UV-VIS spectra of pure compound or similar pure standards and literature data; [b]: dosed with the calibration curve of quercetin-3-*O*-glucoside; [c] dosed with the calibration curve of caftaric acid; [d] dosed with the calibration curve of gallic acid; [e] dosed with the calibration curve of procyanidin B1. The results are reported as mean value ± standard deviation (n = 3).

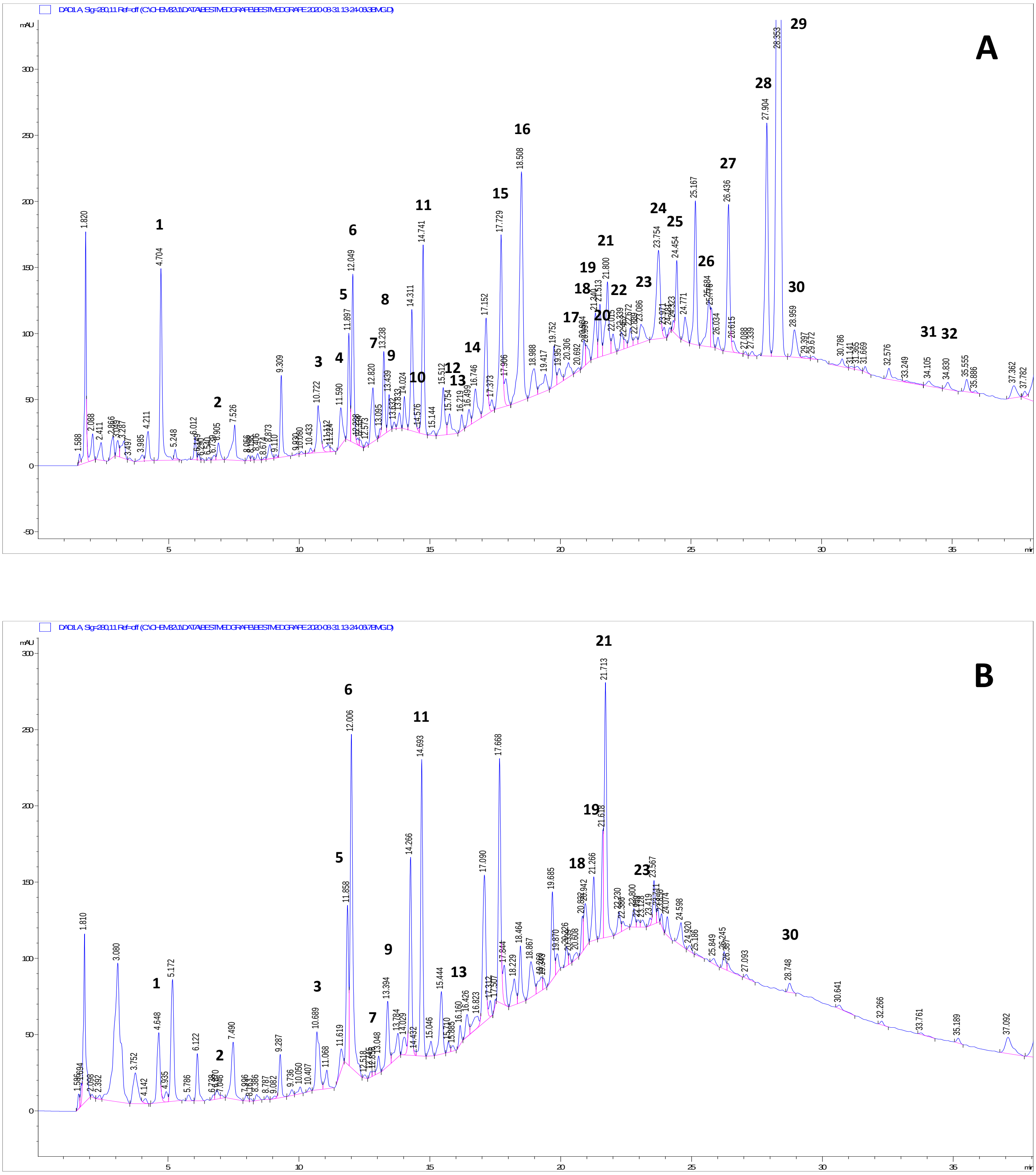

Figure S1. HPLC-DAD chromatograms of Italian Syrah (A) and Tunisian Chardonnay (B) pomaces extracts at λ = 280 nm (for peaks attribution see Table S1 and Table S2). Chromatographic conditions were described in the main manuscript.

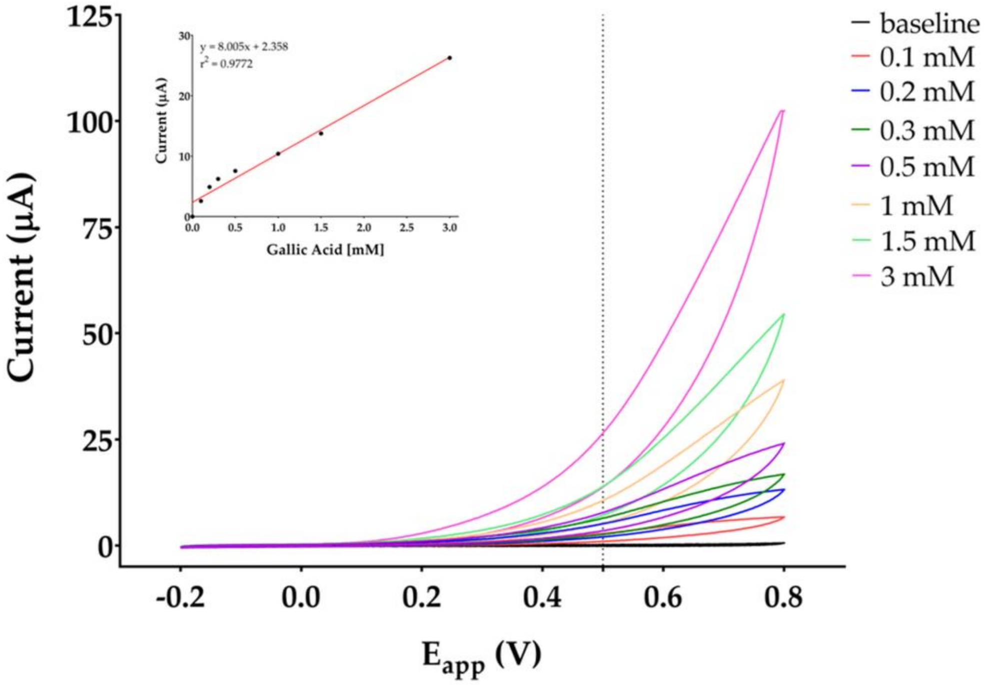

Figure S2. Cyclic voltammograms of growing concentration (from 0.1 to 3 mM) of gallic acid, and the relative calibration curve (inset). CVs were carried out, with a scanned potential range (E_{app}) comprised between –0.2 V and +0.8 V vs. carbon pseudoreference.

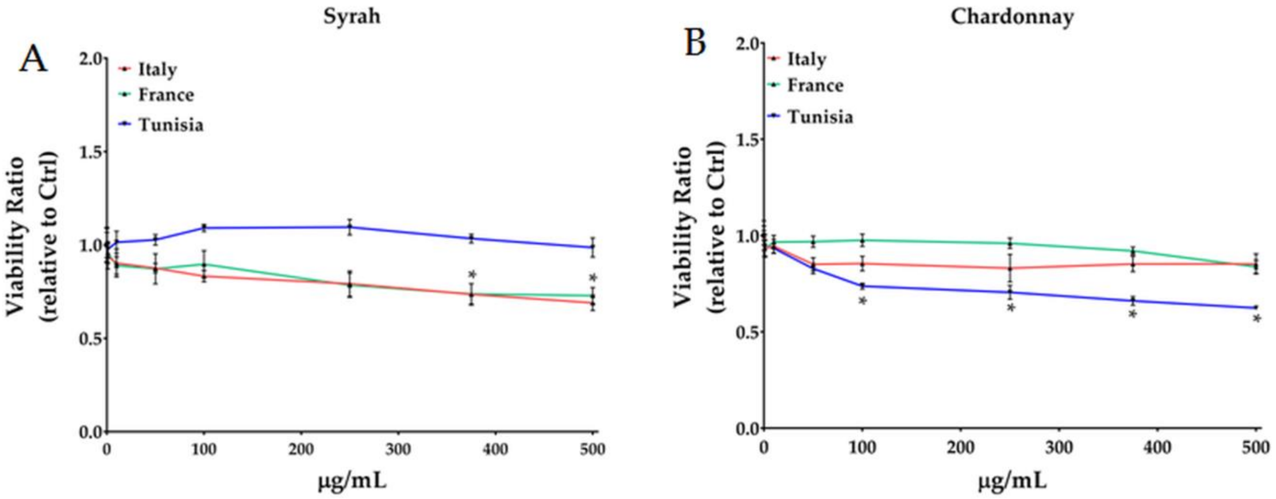

Figure S3. Effects of 24h treatment on fibroblasts with growing concentration (from 1 to 500 µg/mL) of 250 µg/mL of Syrah and Chardonnay pomace extracts

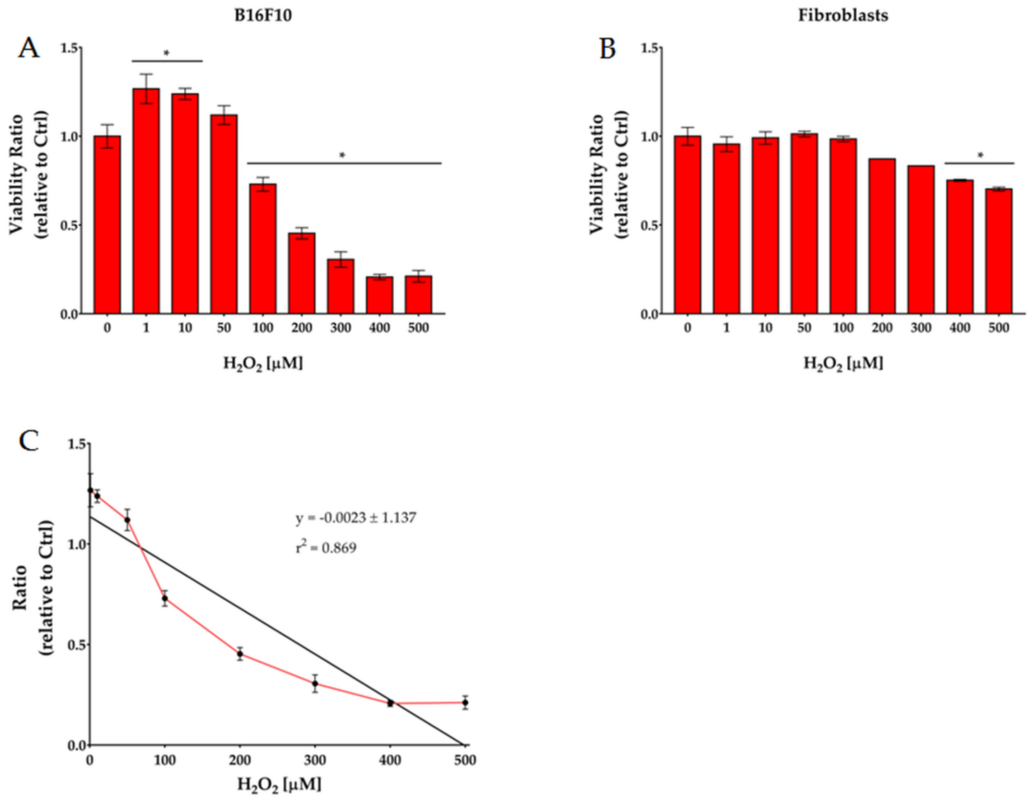

Figure S4. Dose-response viability test with increasing quantity of H_2O_2 on B16F10 cells (Figure S4A) and fibroblasts (Figure S4B), and H_2O_2 calibration curve (1 to 500 μM) (Figure S4C).
